# Neonatal BCG vaccination to prevent asthma: Results from the MIS BAIR randomized controlled trial

**DOI:** 10.1111/pai.70110

**Published:** 2025-06-04

**Authors:** Laure F. Pittet, Emily K. Forbes, Susan Donath, Kate L. Francis, Kaya Gardiner, Katie L. Flanagan, Anne‐Louise Ponsonby, Roy Robins‐Browne, Frank Shann, Mike South, Peter Vuillermin, Dan Casalaz, Nigel Curtis, Nicole L. Messina, the Melbourne Infant Study: BCG for Allergy and Infection Reduction (MIS BAIR) Group

**Affiliations:** ^1^ Infectious Diseases Group Murdoch Children's Research Institute Parkville Victoria Australia; ^2^ Department of Paediatrics The University of Melbourne Parkville Victoria Australia; ^3^ Immunology, Vaccinology, Rheumatology and Infectious Diseases Unit Geneva University Hospitals and Faculty of Medicine Geneva Switzerland; ^4^ Clinical Epidemiology & Biostatistics Unit Murdoch Children's Research Institute Parkville Victoria Australia; ^5^ Department of Research Operations The Royal Children's Hospital Melbourne Parkville Victoria Australia; ^6^ School of Health Sciences University of Tasmania Hobart Tasmania Australia; ^7^ School of Health and Biomedical Science RMIT University Melbourne Victoria Australia; ^8^ Department of Immunology and Pathology Monash University Clayton Victoria Australia; ^9^ Population Allergy Murdoch Children's Research Institute Parkville Victoria Australia; ^10^ The Florey Institute for Neuroscience and Mental Health, University of Melbourne Parkville Victoria Australia; ^11^ Department of Microbiology and Immunology Peter Doherty Institute for Infection and Immunity, the University of Melbourne Parkville Victoria Australia; ^12^ Department of General Medicine The Royal Children's Hospital Melbourne Parkville Victoria Australia; ^13^ School of Medicine Deakin University Geelong Victoria Australia; ^14^ Child Health Research Unit Barwon Health Geelong Victoria Australia; ^15^ Neonatal Intensive Care Unit Mercy Hospital for Women Heidelberg Victoria Australia; ^16^ Department of Infectious Diseases The Royal Children's Hospital Melbourne Parkville Victoria Australia

**Keywords:** asthma, BCG vaccine (*Mycobacterium bovis*), prevention, vaccine non‐specific off‐target effects, wheeze

## Abstract

**Background:**

Asthma has a significant impact worldwide, but prevention strategies remain limited. We aimed to evaluate the efficacy of neonatal BCG vaccination in preventing asthma by modulating early‐life immunity.

**Methods:**

The *Melbourne Infant Study: BCG for Allergy and Infection Reduction* (MIS BAIR) was a phase 3 multicentre randomized controlled trial in Victoria, Australia. Infants were randomly assigned to receive the BCG‐Denmark vaccine or no intervention within 10 days of birth. The incidence of asthma at 5 years of age was estimated using the International Study of Asthma and Allergies in Childhood questions. ClinicalTrial.gov (NCT01906853).

**Results:**

A total of 1272 infants were randomized. The adjusted incidence of asthma was 14.4% in the BCG group compared to 16.0% in the control group (adjusted risk difference [aRD] −1.7 percentage points; 95%CI −7.4, 3.9). Secondary outcomes, including severe asthma and use of preventer medication, showed similar trends, with an aRD of −3.9 (95%CI −7.7, 0.0), and −5.6 (95%CI −10.9, −0.4), respectively, favoring the BCG group. Among participants with one or both parents asthmatic, the rate of asthma was also lower in the BCG group (17.6%) compared with the control group (24.7%; aRD −7.2; 95%CI −15.9, 1.5), although a test for interaction was not significant (*p* = .07).

**Conclusions:**

While the point estimates suggested BCG vaccination might protect against asthma, the wide uncertainty around the estimates means further studies with larger sample sizes are needed to evaluate the long‐term benefits of BCG vaccination beyond its primary indication.

**Key message**

Asthma remains a global health burden with limited preventive strategies. In the Melbourne Infant Study: BCG for Allergy and Infection Reduction (MIS BAIR) randomized controlled trial, neonatal BCG vaccination was associated with a modest reduction in asthma outcomes, particularly severe asthma and in high‐risk children. However, the uncertainty interval around the modest reduction did not indicate a clear, consistent benefit, and therefore further research is needed to clarify BCG's long‐term effects.

## 1 INTRODUCTION

Asthma is a chronic respiratory condition that affects millions of individuals worldwide, particularly children. It is a leading cause of hospitalization and missed school days.^1^ Despite advancements in treatment, asthma remains a significant public health concern due to its persistent symptoms, effect on quality of life, and healthcare costs. Finding effective prevention strategies has therefore become a priority in asthma research.

The Bacillus Calmette‐Guérin (BCG) vaccine, developed to prevent tuberculosis, has been investigated for its potential immunomodulatory effects beyond tuberculosis prevention.^2^, ^3^ Evidence suggests that the BCG vaccine may influence the development of allergic and autoimmune diseases by modulating the immune system.^4^, ^5^ For example, studies indicate that BCG vaccination may skew the immune system towards a Th1‐dominant response, reducing the risk of Th2‐mediated diseases, such as asthma.^6^, ^7^, ^8^ Three RCTs in low‐mortality settings reported a reduction in the risk of eczema in the first year of life following neonatal BCG vaccination, but only in those with an atopic predisposition.^9^, ^10^, ^11^, ^12^ None of the RCTs found a reduction in the risk of wheezing or food allergy following BCG.^13^, ^14^, ^15^ In observational studies, there is conflicting evidence about the effect of BCG vaccination on the risk of allergic disease, with studies reporting a beneficial effect, no effect, or an increased risk.^16^ There is a possible beneficial effect of BCG against asthma,^17^ that needs to be evaluated in a randomized controlled trial.

This randomized controlled trial aimed to evaluate the efficacy of neonatal BCG vaccination in protecting against the development of asthma in children.

## 2 METHODS

The *Melbourne Infant Study: BCG for Allergy and Infection Reduction* (MIS BAIR) was a phase 3 multicentre randomized controlled trial conducted in Victoria, Australia. The study aimed to evaluate the effect of neonatal BCG vaccination to prevent infection, allergic diseases, and asthma in the first years of life. This trial was approved by the Royal Children's Hospital Human Research Ethics Committee (33025), Mercy Health Human Research Ethics Committee (R12‐28) and the ethics committees of all participating hospitals. The parents or guardians of all participants provided signed informed consent before randomization. The trial protocol has been published and is registered on ClinicalTrial.gov (NCT01906853).^18^

Briefly, neonates were randomly allocated to either the BCG group or the control group in a 1:1 ratio using a secure, web‐based system (REDCap®).^19^ Infants in the BCG group received a single 0.05 mL intradermal dose of BCG‐Denmark vaccine (Statens Serum Institute; *Mycobacterium bovis*, Danish strain 1331) over the left deltoid region. Due to the nature of BCG vaccination, which results in a visible scar, families were aware of their group allocation. However, the study staff remained blinded to participant allocation throughout the trial.

The trial involved Part 1 (from birth to 1 year of age) and Part 2 (from one to 5 years of age). At 1 year of age, participants were asked to re‐consent if they wished to continue into Part 2 of the trial. Asthma was evaluated at 5 years of age using validated questions from the *International Study of Asthma and Allergies in Childhood* (ISAAC),^20^ and additional questions on asthma medication. The questionnaire was distributed during Melbourne's strict COVID‐19 lockdown, leading to higher rates of missing data as parents managed work and home schooling. A catch‐up questionnaire was sent post‐lockdown, when participants were 7–10 years of age, to those with missing primary analysis data. Only “no” responses (indicating the absence of the outcome) were considered valid, as “yes” responses could reflect diagnoses outside the study period.

At the end of Part 1, children were invited to a 1‐year visit during which an allergy nurse did a skin prick test (SPT) to a panel of aeroallergens and food allergens as previously reported.^15^ Additional detail is provided in an online data supplement.

### 2.1 Statistical analysis

The primary outcome was the incidence of asthma at 5 years of age, analyzed using a multiple imputation model to handle missing data, in the intention‐to‐treat population. Participants were considered to have asthma if the parents responded “yes” to “Has your child ever had asthma?” at the 5‐years questionnaire. Adjusted risk differences (aRD) were calculated using binary regression adjusted for the randomization stratification factor of birth mode and reported with 95% confidence interval (95% CI), together with estimation of the number‐needed‐to‐treat (NNT) to prevent one case. A sensitivity analysis was performed, disregarding the data collected through the catch‐up questionnaire. Additional adjusted models were estimated to explore the potential heterogeneity of the effect of the intervention,^21^, ^22^ including: asthma in one or both parents, sex, maternal history of BCG vaccination, and (as a post hoc subgroup analysis) allergic sensitisation (defined as a positive SPT at the 1‐year visit). The proportion of participants with asthma (primary outcome) and/or allergic sensitisation was described in four subgroups: neither, one condition, or both (i.e., atopic asthma).

Secondary outcomes were analyzed similarly and included: current (active) asthma, current “wheezing disease” (not necessarily diagnosed as asthma), asthma severity, and use of preventer medication (e.g., fluticasone, ciclesonide, budesonide, montelukast). Severe asthma was defined as having, in the last 12 months, 4 or more attacks of wheezing, one or more nights per week of disturbed sleep, or one episode of speech limitation to one or two words due to wheeze. Further details on outcome definitions and analysis, imputation models, and sample size calculation are available in the statistical analysis plan (Appendix—S1). Briefly, multiple imputation was done by chained equations, producing 50 imputed datasets of the full sample (*N* = 1272), under the missing at random assumption. A single imputation model with all outcome variables included did not converge, so an individual imputation model was performed for the primary outcome and secondary outcomes of current asthma and use of preventer medication. The original sample size (*n* = 1438) was calculated for Part 1 outcomes, rather than for the asthma outcomes. For Part 2, it was estimated that a sample size of 1294 participants would allow the detection of a 6% absolute reduction in the incidence of asthma (from 22% to 16%).^23^ All analyses were done using Stata v18.0 (StataCorp, College Station, Texas).

## 3 RESULTS

**FIGURE 1:**
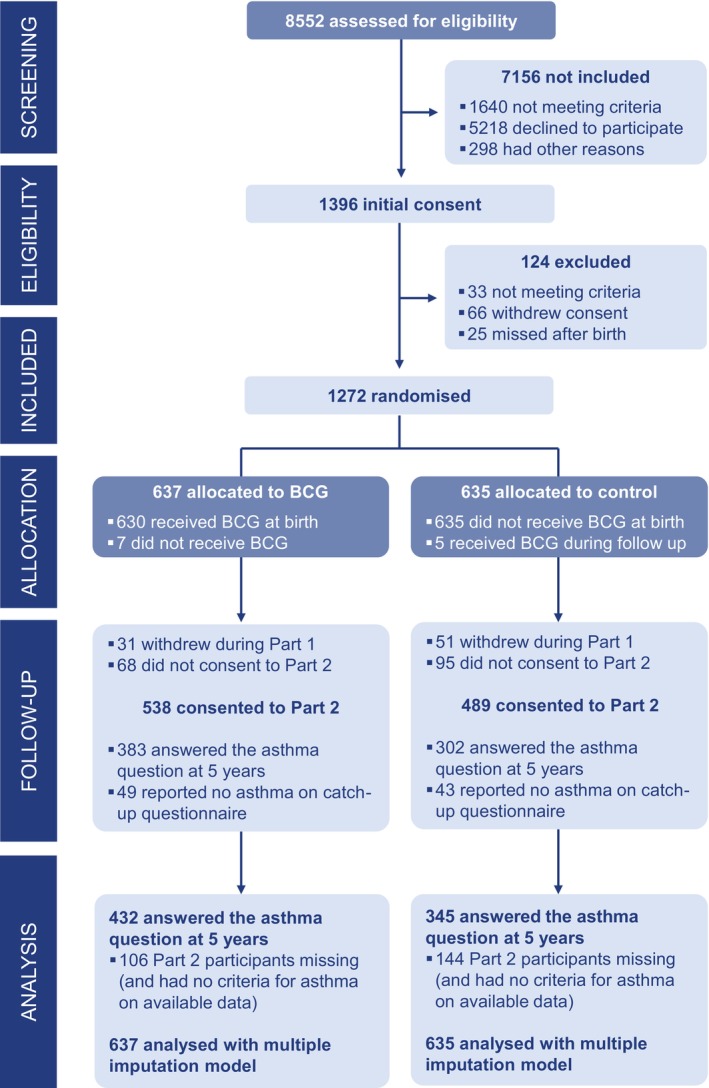
CONSORT diagram. BCG, bacille Calmette‐Guérin.

A total of 1272 infants were enrolled and randomized between August 2013 and September 2016. Recruitment had to cease prematurely due to a worldwide shortage of BCG‐Denmark vaccine. A total of 637 were allocated to the BCG group and 635 to the control group (Figure 1). Their characteristics are presented in Table 1 and Table SE1 (online supplement). Of the 1272 infants participating in Part 1, 1027 consented to continue into Part 2 (80.7%). In total, 777 infants had complete data for the primary outcome of current asthma (76% of Part 2 participants, 61% of all randomized participants): 432 in the BCG group and 345 in the control group; 33% (342/1027) of Part 2 participants had missing data for the primary outcome. Of these, 36% (124/342) completed the catch‐up questionnaire. Characteristics of participants with data available for the primary outcome analysis are presented by group in the Supplementary Table SE1 (online supplement).

**TABLE 1:** Participants characteristics and exposures during first 5 years of life.

	*N*	All participants	BCG	Control
		*n* = 1272	*n* = 637	*n* = 635
Infant factors				
Age at randomization, hours	1272	46.9 (43.3)	46.0 (41.3)	47.8 (45.2)
Sex, female	1272	630 (49.5%)	318 (49.9%)	312 (49.1%)
Birth weight, kg	1272	3.4 (0.5)	3.4 (0.5)	3.4 (0.5)
Gestational age at birth, weeks	1272	39.3 (1.4)	39.4 (1.4)	39.2 (1.4)
Twin pregnancy	1272	21 (1.7%)	11 (1.7%)	10 (1.6%)
Vaginal delivery	1272	812 (63.8%)	406 (63.7%)	406 (63.9%)
Hep B vaccination ≤24 h post randomization	1271	1103 (86.8%)	541 (85.1%)	562 (88.5%)
*Infant ethnicity*	1272			
Caucasian (3–4 grandparents Caucasian)		949 (74.6%)	474 (74.4%)	475 (74.8%)
Asian (3–4 grandparents Asian)		82 (6.4%)	43 (6.8%)	39 (6.1%)
Mixed Caucasian and Asian		62 (4.9%)	32 (5.0%)	30 (4.7%)
Other		179 (14.1%)	88 (13.8%)	91 (14.3%)
*Season of birth*	1272			
Summer		286 (22.5%)	149 (23.4%)	137 (21.6%)
Autumn		362 (28.5%)	177 (27.8%)	185 (29.1%)
Winter		314 (24.7%)	158 (24.8%)	156 (24.6%)
Spring		310 (24.4%)	153 (24.0%)	157 (24.7%)
Maternal factors				
Age at delivery, year	1271	32.6 (4.8)	32.6 (4.8)	32.7 (4.7)
Primipara	1272	686 (53.9%)	355 (55.7%)	331 (52.1%)
Smoking during pregnancy	1269	43 (3.4%)	23 (3.6%)	20 (3.1%)
History of BCG vaccination	1206	318 (26.4%)	159 (26.3%)	159 (26.5%)
*Maternal education*	1269			
No education / up to Year 10		75 (5.9%)	41 (6.5%)	34 (5.4%)
Year 12/trade		340 (26.8%)	165 (26.0%)	175 (27.6%)
University		854 (67.3%)	428 (67.5%)	426 (67.1%)
Familial and environmental factors at birth				
Number of household habitants	1272	2.9 (1.1)	2.9 (1.2)	2.9 (1.1)
Parental history of asthma	1269	577 (45.5%)	298 (46.9%)	279 (44.0%)
Family history of any atopic disease^a^	1271	1049 (82.5%)	529 (83.2%)	520 (81.9%)
Both parents have an atopic disease^a^	1269	386 (30.4%)	192 (30.2%)	194 (30.6%)
Smoker living in the house during pregnancy	1269	222 (17.5%)	105 (16.5%)	117 (18.5%)
Exposure during first 5 years of life				
Family had a cat at some point	1070	396 (37.0%)	201 (36.4%)	195 (37.6%)
Family had a dog at some point	1110	583 (52.5%)	294 (52.3%)	289 (52.7%)
Smoke exposure during first 5 years of life	1272	279 (21.9%)	138 (21.7%)	141 (22.2%)
Positive SPT at 1 year visit (allergic sensitisation)	1066	224 (21.0%)	126 (22.5%)	98 (19.3%)

*Note*: Categorical variables are reported as number (%), continuous variables are reported as mean (standard deviation).

Abbreviations: BCG, bacille Calmette‐Guérin; h, hours; SPT, skin prick test; y, years.

^a^ Any of eczema, hay fever, asthma.

**FIGURE 2:**
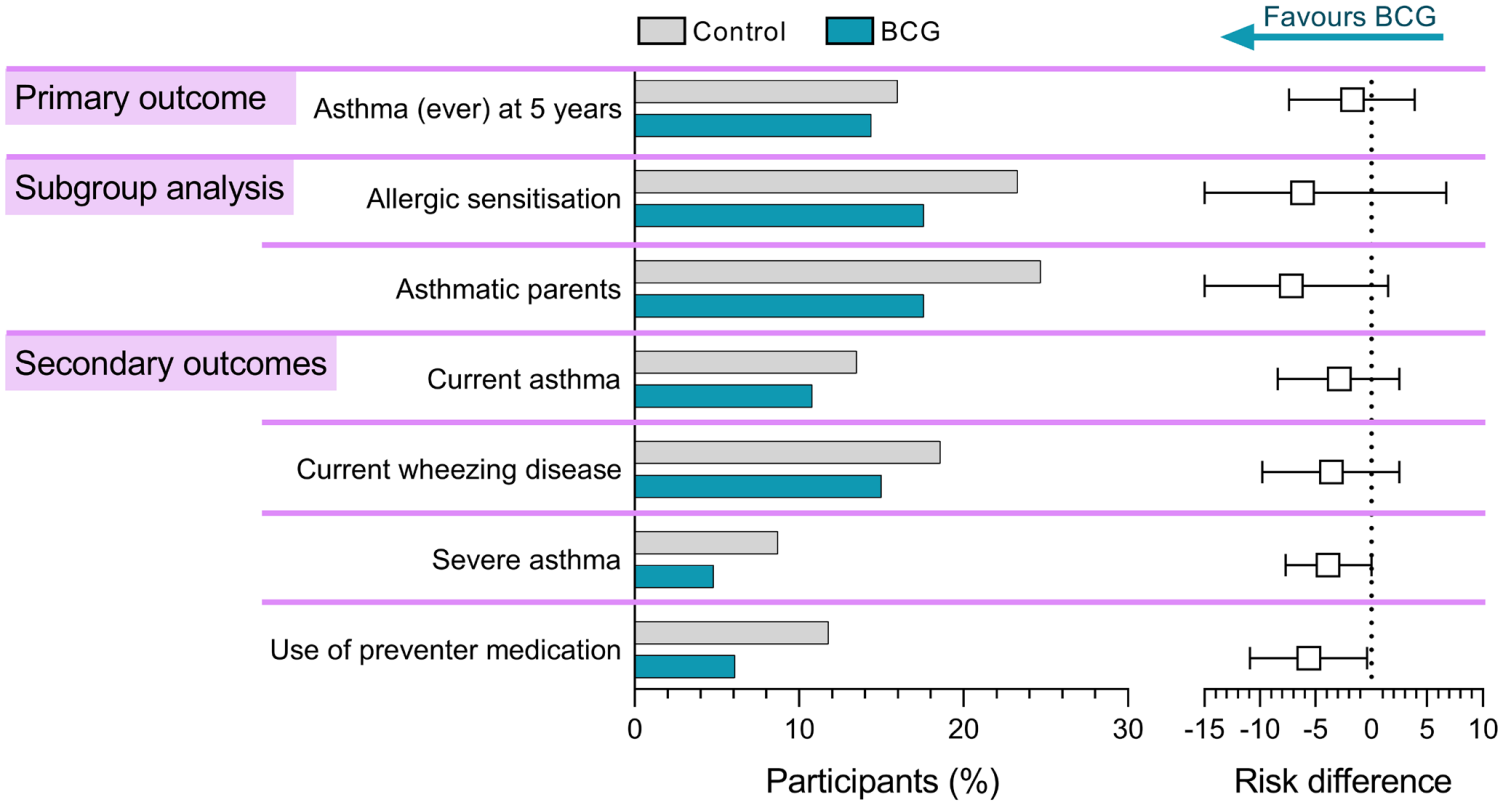
Difference in presence and severity of asthma between BCG and control groups, assessed at 5 years of age. Left: Bars represent the proportion of participants fulfilling the criteria for the primary and secondary outcomes. Right: Squares represent adjusted risk differences for the multiple imputation models, with error bars depicting 95% CI. Allergic sensitization was defined as positive skin prick test at the 1‐year visit (post hoc subgroup analysis).

After multiple imputation, the adjusted incidence of ever having asthma was 14.4% in the BCG vaccine group and 16.0% in the control group, with an aRD of −1.7 percentage points (95% CI, −7.4 to 3.9; Table 2 and Figure 2). Similar results were found for the adjusted proportion of current asthma (10.8% in the BCG group vs. 13.5% in the control group, aRD of −2.9 percentage points, 95% CI −8.4 to 2.5), current wheezing disease (15.0% vs. 18.6%, aRD −3.6 percentage points, 95% CI −9.8 to 2.5), severe asthma at 5 years of age (4.8% vs. 8.7%, aRD −3.9 percentage points, 95% CI −7.7 to 0.0), and use of prevention medication at 5 years of age (6.1% vs. 11.8% aRD −5.6 percentage points, 95% CI −10.9 to −0.4; Table 2 and Figure 2). A sensitivity analysis excluding data from the catch‐up questionnaire had similar results and is presented alongside the complete case analysis in the online supplement.

**TABLE 2:** Asthma at 5 years of age.

	BCG	Control	Adjusted risk difference (95% CI)^a^	NNT
	*n* = 637	*n* = 635		
Primary outcome				
Asthma (ever) by 5 years of age	14.4%	16.0%	−1.7% (−7.4% to +3.9%)	63
Secondary outcomes				
Current asthma, at 5 years of age	10.8%	13.5%	−2.9% (−8.4% to +2.5%)	37
Current wheezing disease, at 5 years of age	15.0%	18.6%	−3.6% (−9.8% to +2.5%)	28
Severe asthma, at 5 years of age	4.8%	8.7%	−3.9% (−7.7% to 0.0%)	26
Use of prevention medication, at 5 years of age	6.1%	11.8%	−5.6% (−10.9% to −0.4%)	18
Subgroup analysis (for primary outcome)				
Female	9.0%	13.1%	−4.1% (−10.8% to +2.6%)	24
Male	19.9%	18.8%	+0.8% (−8.2% to +9.7%)	NA
One or two parents with asthma	17.6%	24.7%	−7.2% (−15.9% to 1.5%)	14
No parent with asthma	11.7%	9.0%	+2.6% (−4.4% to 9.5%)	NA
Born to BCG‐naïve mother	13.8%	16.0%	−2.2% (−9.1% to +4.7%)	45
Born to previously BCG‐vaccinated mother	16.2%	16.3%	−0.5% (−10.6% to +9.5%)	NA
Allergic sensitisation	17.6%	23.3%	−6.2% (−19.2% to +6.7%)	18
No allergic sensitisation	11.7%	12.3%	−0.5% (−6.0% to +5.0%)	167

Abbreviations: BCG, bacille Calmette‐Guérin; CI, confidence interval; NA, not applicable; NNT, number needed to treat (number needed to vaccinate with BCG to prevent 1 case of asthma); y, years.

^a^ Adjusted for stratification mode of delivery.

**FIGURE 3:**
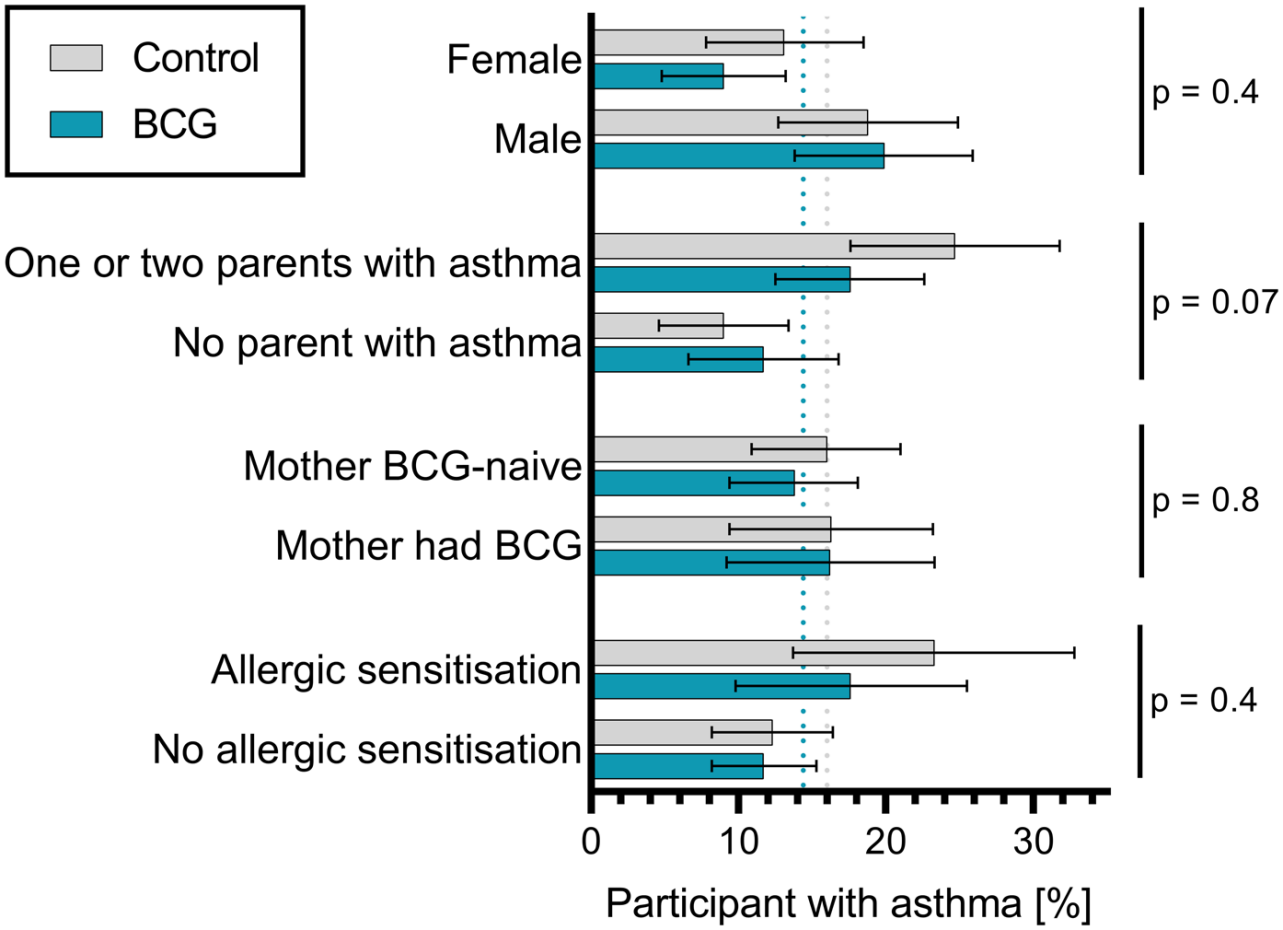
Subgroup analysis of incidence of asthma at 5 years of age. Bars represent the proportion of participants fulfilling the criteria for the primary outcome of asthma in prespecified subgroups; error bars depict 95% CI. The vertical dotted lines represent the proportion of participants with asthma in the whole BCG (blue) and control (gray) groups. *p*‐values are for the interaction term (BCG status by variable). Allergic sensitisation was defined as a positive skin prick test at the 1‐year visit (post hoc subgroup analysis).

Subgroup analyses of the imputed data revealed no evidence of interaction (Table 2 and Figure 3). Children born to parents with asthma were more likely to have asthma. In this high‐risk group, the rate of asthma was lower in the BCG group (17.6%) compared with the control group (24.7%; aRD −7.2 percentage points, 95% CI −15.9 to 1.5, interaction test *p* = .07). In the post hoc analysis, children with allergic sensitisation were overall more likely to have asthma, with a lower rate in the BCG group (17.6%) compared with the control group (23.3%; aRD −6.2 percentage points, 95% CI −19.2 to 6.7, interaction test *p* = .4). Comparing the presence of asthma with or without allergic sensitisation showed that the decrease in the prevalence of asthma in the BCG group was mostly observed among children with allergic sensitisation (“atopic asthma”), however, small numbers precluded a 4 subgroups analysis (interaction test *p* = .4, Figure SE1 and Table SE2). Males were more likely to have asthma than females. Among females, the rate of asthma was lower in the BCG group (9.0%) compared with the control group (13.1%; aRD −4.1 percentage points, 95% CI −10.8 to 2.6, interaction test *p* = .4). Subgroup analysis according to maternal history of BCG vaccination showed similar asthma rates across groups (interaction test *p* = .8, Table 2 and Figure 3).

## 4 DISCUSSION

In this RCT, we found neonatal BCG vaccination had minimal impact on the incidence of asthma at 5 years of age overall but was associated with reductions in more severe asthma and asthma among high‐risk infants. The proportion of affected children was consistently lower in the BCG group for the primary and secondary outcomes. However, given the large uncertainty around the point estimates, the findings may not represent a true protective effect.

While many studies in mice have demonstrated a clear preventive and therapeutic effect of BCG vaccination against allergy and asthma, the evidence in humans is less consistent.^5^ There is some evidence that neonatal BCG vaccination reduces the risk of eczema in the first year of life.^24^ Epidemiological, cohort, case–control, and cross‐sectional studies on the effect of BCG vaccination on asthma and other allergic diseases have been summarized in several meta‐analyses.^25^, ^26^, ^27^, ^28^, ^29^, ^30^ The most recent estimate, restricted to higher‐quality cohort studies, suggested a 25% decrease in the risk of asthma in BCG‐vaccinated children.^30^ However, methodological issues have been raised in most of the studies, and RCTs are needed.^5^ Previous systematic reviews and meta‐analyses have included RCTs reporting on infant wheeze, assessed at 13 or 18 months of age,^9^, ^31^ and concluded there was no significant effect on asthma.^29^ As asthma cannot be reliably diagnosed before the age of 5 (as most wheezing pre‐schoolers do not progress to asthma),^32^ these RCTs cannot provide conclusive evidence about childhood asthma. Therefore, to our knowledge, this is the first RCT to assess the effect of neonatal BCG vaccination on childhood asthma.

In our study, children born to asthmatic parents had a higher risk of developing asthma; among them, we observed a 7.2 percentage point lower risk in those who received BCG, compared to the non‐vaccinated controls. This corresponds to a NNT of 14 to prevent one case, although the interaction test was not statistically significant (*p* = .07). Early life allergic sensitisation is associated with asthma,^33^ and atopic children are overrepresented in those with more severe asthma.^34^ We therefore performed a post hoc subgroup analysis, which showed that, in children with allergic sensitisation, the risk of asthma was 6.2 percentage points lower in BCG‐vaccinated children compared with controls. This corresponds to a NNT of 18 to prevent one case, although the interaction test was not statistically significant (*p* = .4). This is consistent with a previous report^35^ and other trials suggesting that the off‐target effect of BCG vaccination might only be clinically important when the individual risk is high or for protection against severe diseases. For example, in a meta‐analysis, BCG vaccination decreased the risk of eczema predominantly in children with an atopic predisposition, and a larger effect was observed with an increased predisposition (i.e., no versus one versus two atopic parents).^24^ The beneficial effects of BCG appear to be strongest for severe diseases. In high‐mortality settings, neonatal BCG vaccination‐induced protection against unrelated infections and all‐cause mortality,^36^, ^37^ a finding that was not replicated in low‐mortality settings.^38^, ^39^ This may explain why our data suggest a more substantial beneficial effect of neonatal BCG in preventing severe asthma and the use of prevention medication (which is only prescribed to children with more severe asthma).

A sex‐differential effect was observed, with the effect of BCG vaccination being possibly greater in girls, although the interaction test was not statistically significant. Sex‐based differences in immune responses are well described, and there is a sex‐differential predisposition in autoimmune disorders.^40^, ^41^, ^42^ As reported previously, boys had a higher incidence of asthma compared to girls.^1^, ^21^, ^22^, ^43^ Sex‐differential effects have been observed for off‐target effects of vaccination,^3^, ^44^, ^45^, ^46^ including in immunological studies of participants in this trial.^47^, ^48^ The potential mechanisms underlying these differences remain uncertain; sex chromosomes and sex hormones could both play a role.^44^

We did not find any influence of maternal history of BCG administration on the prevention of asthma. The presence of a BCG scar in the mother has been reported to enhance the off‐target effects of BCG in infants living in both high^49^, ^50^ and low‐mortality settings^38^ as well as in immunological studies.^51^ In our trial, we previously observed that BCG‐vaccinated participants born to a BCG‐naïve mother had a greater protective effect against infant eczema,^52^ and that BCG‐vaccinated participants born to a BCG‐vaccinated mother had a higher risk of atopic sensitization at 1 year of life.^15^ As many BCG‐vaccinated mothers were from Asia whereas BCG‐naïve mothers were mostly Caucasian, the results might just reflect the disproportionally high risk of atopic disease reported in second‐generation East Asian immigrants in Australia.^53^

Our trial has several limitations, including the inability to blind participants to treatment allocation in BCG trials, the lower‐than‐anticipated recruitment numbers, reliance on parent‐reported outcomes, and missing data, partly due to the COVID‐19 pandemic. In addition, we tested only a maternal history of BCG vaccination rather than the more accurate presence of a BCG scar in the mother. To address missing data, we used a catch‐up questionnaire and multiple imputation, with sensitivity analyses that validated the approach. Asthma was defined using the ISAAC questionnaire, recognized and used in epidemiological studies, though it may perform less well than measures such as clinician interviews to determine asthma status. Misclassification in our primary outcomes may have diluted the beneficial effect of BCG evident among children with more clearly defined, severe disease. Several secondary outcomes were explored to gain a more comprehensive understanding of the effect of BCG vaccination on asthma. However, we acknowledge that these outcomes were not assessed in a predefined order, and no correction for multiple testing was applied, increasing the risk of spurious findings. The subgroup analysis on allergic sensitisation was not pre‐specified in the original study design; as a post hoc analysis, the findings should be interpreted with caution. Finally, the small difference in the primary outcome between study groups may be attributed to residual confounding from the open‐label intervention. Our trial strengths include the inclusion of clinically relevant questions on asthma severity and the use of preventer medication. These questions may be the most reliable indicators of clinically significant asthma and were the only outcomes that showed statistically significant differences between the two groups; however, these results are based on a small number of cases and should be interpreted cautiously. Another strength resides in this trial being an RCT and having a long follow‐up (5 years) enabling the assessment of asthma that is more likely to be true asthma rather than transient preschool wheezing.

Overall, while our study did not find a clinically significant protective effect of neonatal BCG vaccination on asthma incidence, our results suggest any potential beneficial effect of neonatal BCG vaccination to prevent severe asthma is predominantly in high‐risk children with one or two asthmatic parents. The potential benefits of BCG vaccination must always be carefully weighed against its risks, the most severe being disseminated BCG infection in infants with rare inborn errors of immunity. As previously reported, there were no safety concerns following BCG vaccination in the MIS BAIR trial.^15^ Further research with larger sample sizes and possibly restricted to high‐risk individuals is necessary to confirm our results and explore the potential long‐term benefits of the BCG vaccine. Our findings contribute to the growing body of literature evaluating the broader health impacts of vaccinations beyond their primary targets.

## AUTHOR CONTRIBUTIONS

N.C. was the lead investigator and responsible for study conception, design, and funding acquisition. N.C. and S.D. developed the final scientific protocol and ethics application, and all other authors provided critical evaluation and revision. K.G. co‐ordinated, and N.C., D.C., P.V., and N.L.M. were involved in implementation. E.F. developed, and L.F.P., N.C., N.L.M., S.D., M.S., and A.L.P. contributed to the statistical analysis plan. E.K.F. led, N.L.M. supervised, and L.F.P. contributed to data cleaning and preparation. K.L.Fr led, and S.D. supervised statistical analysis. L.F.P. drafted the manuscript and co‐ordinated manuscript preparation and revision. All authors provided critical evaluation and revision of the manuscript.

## FUNDING INFORMATION

MIS BAIR was funded by the National Health and Medical Research Council (NHMRC) of Australia (GNT 1051228; 1,099,676), The University of Melbourne, RCH Foundation, and the Murdoch Children's Research Institute. LFP is supported by the Swiss National Science Foundation Early Postdoc Mobility grant (P2GEP3_178155) and Ambizione grant (PZ00P3–209,050). NC is supported by a National Health and Medical Research Council (NHMRC) Investigator Grant (GNT1197117).

## CONFLICT OF INTEREST STATEMENT

The authors declare no conflicts of interest.

## PEER REVIEW

The peer review history for this article is available at https://www.webofscience.com/api/gateway/wos/peer‐review/10.1111/pai.70110.

## Supporting information


**Data S1.** Supporting Information.

**Data S2.** Supporting Information.
