# Supplementary material for: Neonatal BCG vaccination to prevent asthma: Results from the MIS BAIR randomized controlled trial
Source: Pediatr Allergy Immunol. 2025 Jun 4;36(6):e70110. doi: 10.1111/pai.70110 (PMC12136015; doi:10.1111/pai.70110)
Supplement: Supplementary file 1 — Data S1. Supporting Information. [file PAI-36-e70110-s001.docx]

**Online Data Supplement**

**Neonatal BCG vaccination to prevent asthma: results from the MIS BAIR randomised controlled trial**

Laure F. Pittet, Emily K. Forbes, Susan Donath, Kate L Francis, Kaya Gardiner, Katie L. Flanagan, Anne-Louise Ponsonby, Roy Robins-Browne, Frank Shann, Mike South, Peter Vuillermin, Dan Casalaz, Nigel Curtis^*^, Nicole L. Messina^*^, on behalf of the Melbourne Infant Study: BCG for Allergy and Infection Reduction (MIS BAIR) Group.

* Co-last authors, equal contribution.

**Content**

- Supplement to Methods
- Supplementary Table E1
- Supplementary Table E2
- Supplementary Figure E1
- Sensitivity analysis and complete case analysis
- Multiple imputation variables
- Statistical analysis plan

**Supplement to Methods**

**Study Design and Regulation**

The *Melbourne Infant Study: BCG for Allergy and Infection Reduction* (MIS BAIR) was a phase 3 multicentre randomised controlled trial conducted in Victoria, Australia. The study aimed to evaluate the effect of neonatal BCG vaccination to prevent infection, allergic diseases, and asthma in the first years of life. The trial protocol has been published and is registered on ClinicalTrial.gov (NCT01906853).^18^

This trial complied with the NHMRC National Statement on Ethical Conduct in Human Research (2007) and the Note for Guidance on Good Clinical Practice (CPMP/ICH-135/95). It was approved by the Royal Children's Hospital Human Research Ethics Committee (33025) Mercy Health Human Research Ethics Committee (R12-28) and the ethics committees of all participating hospitals. A multidisciplinary and independent data and safety monitoring committee oversaw patient safety and data collection.

**Population**

Infants were eligible if they were born after 32 weeks of gestation, weighed over 1500 grams at birth, were clinically stable, had no sibling in the trial, and had no indications for or contraindications to BCG vaccination. The study involved Part 1 (from birth to one year of age) and Part 2 (from one to five years of age). At 1 year of age, participants were asked to re-consent if they wished to continue into Part 2 of the study. Asthma was evaluated at 5 years of age, during Part 2.

**Randomisation and Blinding**

Participants were randomly allocated to either the BCG group or the control group in a 1:1 ratio using a secure, web-based system (REDCap®).^19^ The randomisation sequence was generated by an independent statistician and utilised random permuted blocks of variable sizes. Stratification was done by mode of delivery (vaginal vs. caesarean), a plurality (singleton vs. twin), and recruitment site to ensure balanced allocation. Stratification by mode of delivery was done to address its potential impact on the infant gut microbiome and the development of atopy.^20^

**Intervention**

Infants in the BCG group received a single 0.05 ml intradermal dose of BCG-Denmark vaccine (Statens Serum Institute; *Mycobacterium bovis*, Danish strain 1331) over the left deltoid within 24 hours of randomisation and before 10 days of age. Due to the nature of BCG vaccination, which results in a visible scar, families were aware of their group allocation. However, the study staff remained blinded to participant allocation throughout the trial.

**Data Collection**

Baseline data were obtained at inclusion and included demographics, pregnancy, and perinatal information. Parents completed regular follow-up questionnaires throughout the trial: every 3 months during Part 1 and every 6 months during Part 2. The 5-year questionnaire included validated questions from the *International Study of Asthma and Allergies in Childhood* (ISAAC)^21^ to assess the presence and severity of asthma, and additional questions on asthma medication. The distribution of this questionnaire coincided with the COVID-19 pandemic, during which Melbourne experienced a strict lockdown; as many parents had to work from home while home schooling, this increased the amount of missing data. A catch-up questionnaire was sent after the after the lifting of lockdown to participants with missing data for the primary analysis questions, when they were seven to ten years old, to identify those who had not had the outcome established. Only ‘no’ responses (i.e. indicating the participant had never had the outcome) were considered as valid data. ‘Yes’ responses were not considered, as the diagnosis could have occurred after the study period.

**Skin prick test**

At the end of Part 1, children were invited to a 1-year visit during which an allergy nurse did a skin prick test (SPT) to a panel of aeroallergens and food allergen as previously reported.^15^ Aeroallergens were house dust mite (*Dermatophagoides pteronyssinus*, ALK, Spain), cat hair (Hollister-Stier Laboratories, WA or Greer Laboratories), dog (Hollister-Stier Laboratories or Greer Laboratories) and rye grass (Hollister-Stier Laboratories). Core food allergens for SPT were cow's milk (ALK, Spain), raw egg white (ALK, Spain), peanut (ALK, Spain), cashew nut (Greer Laboratories, Stallergenes), hazelnut (ALK, Spain) sesame (ALK, USA), and shellfish (ALK, Spain). Histamine (ALK, Spain) was the positive control, and saline (ALK, Spain) was the negative control. Allergens were applied using a QUINTIP device (Hollister-Stier Laboratories, WA), and wheal size (mean diameter) was measured at 15 min. Participants were considered sensitised for an allergen if the wheal size was ≥2 mm greater than the negative control, and having allergic sensitisation if sensitised to ≥1 allergen. Participants were considered negative if all allergen wheals were less than or equal to the negative control, and the positive control was ≥1 mm.

**Statistical Analysis**

The primary outcome was the incidence of asthma at 5 years of age, analysed using a multiple imputation model to handle missing data, in the intention-to-treat population. Participants were considered to have asthma if the parents responded ‘yes’ to ‘Has your child ever had asthma?’ at the 5-years questionnaire. Adjusted risk differences (aRD) were calculated using binary regression adjusted for the randomisation stratification factor of birth mode and reported with 95% confidence interval (95% CI). The two other stratification factors (site and plurality) were not included as adjustments because 98% of infants were singleton and 95% were from the same site. A sensitivity analysis was performed, disregarding the data collected through the catch-up questionnaire. Additional adjusted models were estimated to explore the potential heterogeneity of the effect of the intervention,^22,23^ including: asthma in one or both parents, sex, maternal history of BCG vaccination, and (as a post hoc subgroup analysis) allergic sensitisation (defined as a positive SPT at the 1-year visit). The proportion of participants in the BCG group and the control group with asthma (as defined by the primary outcome) and/or allergic sensitisation were described, categorised as 4 subgroups: neither, one of the conditions, or both.

Secondary outcomes were analysed similarly and included: current (active) asthma, current “wheezing disease” (not necessarily diagnosed as asthma), asthma severity, and use of preventer medication (e.g., fluticasone, ciclesonide, budesonide, montelukast). Severe asthma was defined as having, in the last 12 months, 4 or more attacks of wheezing, one or more nights per week of disturbed sleep, or one episode of speech limitation to one or two words due to wheeze. Further details on outcome definitions and analysis are available in the statistical analysis plan (appendix). All analyses were done using Stata v18.0 (StataCorp, College Station, Texas).

The original sample size estimate (n=1438) was calculated for Part 1 outcomes rather than for the asthma outcomes. The Asthma in Australia 2011 report, indicated that for children 4-5 years of age 15.4% have ‘current asthma’ and 22% have ‘asthma ever’.^24^ Assuming that 90% of participants would remain in the study to 5 years (n=1294), it was estimated that this sample size would allow detection of an absolute reduction in current asthma of 5.5% (from 15% to 9.5%) and an absolute reduction in ‘asthma ever’ of 6% (from 22% to 16%)^18^. The final recruited sample size in Part 1 was 1272, due to the worldwide shortage of BCG vaccine, and a total of 1027 of these consented to Part 2.

As some families did not participate in Part 2, and some dropped out, the proportion of 1272 children with missing data for the primary and secondary outcomes ranged from 39% to 46%. Multiple imputation by chained equations was conducted to handle missing data, producing 50 imputed datasets of the full sample N=1272, under the missing at random assumption. A single imputation model with all outcome variables included did not converge, so an individual imputation model was performed for the primary outcome and secondary outcomes of current asthma and use of preventer medication. The secondary outcomes of wheeze disorder and asthma severity were estimated in a single conditional imputation model because the severity variable was conditional on wheeze being present. The results reported here are from the multiple imputation analysis. Further information on the imputation models is available in the appendix.

**Supplementary Table E1: Participants characteristics and exposure during first years of life, by group and data availability**

|  | All participants | Complete primary outcome asthma data | | Missing primary outcome asthma data | |
| --- | --- | --- | --- | --- | --- |
|  | Total | BCG | Control | BCG | Control |
|  | N=1272 | N=432 | N=345 | N=205 | N=290 |
| Maternal factors |  |  |  |  |  |
| Maternal age at delivery (N=1271) | mean=32.6  (sd 4.8) | mean=33.1 (sd 4.3) | mean=33.5  (sd 4.4) | mean=31.5 (sd 5.5) | mean=31.7 (sd 4.9) |
| Primipara at the time of participant's birth (N=1272) | 686/1272 (53.9%) | 237/432 (54.9%) | 193/345 (55.9%) | 118/205 (57.6%) | 138/290 (47.6%) |
| Maternal antibiotic use in 3rd trimester (excl labour) (N=1266) | 169/1266 (13.3%) | 57/431 (13.2%) | 47/344 (13.7%) | 29/203 (14.3%) | 36/288 (12.5%) |
| Maternal birth country / region (N=1272) |  |  |  |  |  |
| Africa | 21/1272 (1.7%) | 8/432 (1.9%) | 2/345 (0.6%) | 5/205 (2.4%) | 6/290 (2.1%) |
| Australia | 923/1272 (72.6%) | 317/432 (73.4%) | 251/345 (72.8%) | 150/205 (73.2%) | 205/290 (70.7%) |
| Continental Europe | 42/1272 (3.3%) | 17/432 (3.9%) | 8/345 (2.3%) | 5/205 (2.4%) | 12/290 (4.1%) |
| East & South East Asia | 85/1272 (6.7%) | 24/432 (5.6%) | 32/345 (9.3%) | 13/205 (6.3%) | 16/290 (5.5%) |
| Middle East | 20/1272 (1.6%) | 5/432 (1.2%) | 2/345 (0.6%) | 5/205 (2.4%) | 8/290 (2.8%) |
| North America | 18/1272 (1.4%) | 8/432 (1.9%) | 3/345 (0.9%) | 3/205 (1.5%) | 4/290 (1.4%) |
| Oceania excluding Australia | 41/1272 (3.2%) | 11/432 (2.5%) | 14/345 (4.1%) | 8/205 (3.9%) | 8/290 (2.8%) |
| South America | 14/1272 (1.1%) | 4/432 (0.9%) | 4/345 (1.2%) | 3/205 (1.5%) | 3/290 (1.0%) |
| South Asia | 38/1272 (3.0%) | 10/432 (2.3%) | 8/345 (2.3%) | 7/205 (3.4%) | 13/290 (4.5%) |
| UK or Ireland | 70/1272 (5.5%) | 28/432 (6.5%) | 21/345 (6.1%) | 6/205 (2.9%) | 15/290 (5.2%) |
| Maternal BMI pre-pregnancy (N=737) | mean=25.2 (sd 5.4) | mean=25.3 (sd 5.3) | mean=25.5 (sd 5.9) | mean=24.4 (sd 4.8) | mean=24.8 (sd 5.0) |
| Maternal weight gain in pregnancy (kgs) (N=742) | mean=12.3 (sd 7.3) | mean=12.5 (sd 7.1) | mean=11.4 (sd 7.4) | mean=12.7 (sd 7.6) | mean=12.8 (sd 7.0) |
| Maternal probiotic use during pregnancy (N=1262) | 208/1262 (16.5%) | 72/431 (16.7%) | 66/344 (19.2%) | 26/202 (12.9%) | 44/285 (15.4%) |
| Mother vitamin D under 50nmol/L during pregnancy (N=1118) | 366/1118 (32.7%) | 113/375 (30.1%) | 92/305 (30.2%) | 71/180 (39.4%) | 90/258 (34.9%) |
| Maternal vitamin D supplements taken during pregnancy (N=1267) | 859/1267 (67.8%) | 297/431 (68.9%) | 231/345 (67.0%) | 136/201 (67.7%) | 195/290 (67.2%) |
| Mother education level (N=1269) |  |  |  |  |  |
| No education / up to year 10 | 75/1269 (5.9%) | 14/430 (3.3%) | 6/345 (1.7%) | 27/204 (13.2%) | 28/290 (9.7%) |
| Year 12 / trade | 340/1269 (26.8%) | 98/430 (22.8%) | 75/345 (21.7%) | 67/204 (32.8%) | 100/290 (34.5%) |
| University | 854/1269 (67.3%) | 318/430 (74.0%) | 264/345 (76.5%) | 110/204 (53.9%) | 162/290 (55.9%) |
| Maternal smoking during pregnancy (N=1269) | 43/1269 (3.4%) | 8/431 (1.9%) | 5/345 (1.4%) | 15/203 (7.4%) | 15/290 (5.2%) |
| Maternal BCG vaccination (N=1206) | 318/1206 (26.4%) | 105/408 (25.7%) | 89/331 (26.9%) | 54/197 (27.4%) | 70/270 (25.9%) |
| Mother GBS positive during pregnancy (N=1272) | 198/1272 (15.6%) | 63/432 (14.6%) | 64/345 (18.6%) | 27/205 (13.2%) | 44/290 (15.2%) |
| Paternal factors |  |  |  |  |  |
| Paternal birth country / region (N=1255) |  |  |  |  |  |
| Africa | 33/1255 (2.6%) | 11/429 (2.6%) | 4/339 (1.2%) | 9/201 (4.5%) | 9/286 (3.1%) |
| Australia | 925/1255 (73.7%) | 326/429 (76.0%) | 251/339 (74.0%) | 137/201 (68.2%) | 211/286 (73.8%) |
| Continental Europe | 38/1255 (3.0%) | 11/429 (2.6%) | 11/339 (3.2%) | 12/201 (6.0%) | 4/286 (1.4%) |
| East & South EastAsia | 52/1255 (4.1%) | 21/429 (4.9%) | 15/339 (4.4%) | 10/201 (5.0%) | 6/286 (2.1%) |
| Middle East | 23/1255 (1.8%) | 4/429 (0.9%) | 5/339 (1.5%) | 6/201 (3.0%) | 8/286 (2.8%) |
| North America | 12/1255 (1.0%) | 1/429 (0.2%) | 6/339 (1.8%) | 2/201 (1.0%) | 3/286 (1.0%) |
| Oceania excluding Australia | 43/1255 (3.4%) | 10/429 (2.3%) | 15/339 (4.4%) | 7/201 (3.5%) | 11/286 (3.8%) |
| South America | 8/1255 (0.6%) | 2/429 (0.5%) | 2/339 (0.6%) | 0/201 (0.0%) | 4/286 (1.4%) |
| South Asia | 38/1255 (3.0%) | 10/429 (2.3%) | 8/339 (2.4%) | 8/201 (4.0%) | 12/286 (4.2%) |
| UK or Ireland | 83/1255 (6.6%) | 33/429 (7.7%) | 22/339 (6.5%) | 10/201 (5.0%) | 18/286 (6.3%) |
| Paternal age at delivery (N=1239) | mean=34.5 (sd 5.5) | mean=35.0 (sd 5.2) | mean=35.2 (sd 5.2) | mean=33.4 (sd 6.2) | mean=33.6 (sd 5.8) |
| Birth factors |  |  |  |  |  |
| Mode of delivery (binary) (N=1272) |  |  |  |  |  |
| C-section | 460/1272 (36.2%) | 160/432 (37.0%) | 117/345 (33.9%) | 71/205 (34.6%) | 112/290 (38.6%) |
| Vaginal | 812/1272 (63.8%) | 272/432 (63.0%) | 228/345 (66.1%) | 134/205 (65.4%) | 178/290 (61.4%) |
| Emergency C-section (N=1272) | 230/1272 (18.1%) | 83/432 (19.2%) | 56/345 (16.2%) | 37/205 (18.0%) | 54/290 (18.6%) |
| Season of birth (N=1272) |  |  |  |  |  |
| Summer | 286/1272 (22.5%) | 97/432 (22.5%) | 79/345 (22.9%) | 52/205 (25.4%) | 58/290 (20.0%) |
| Autumn | 362/1272 (28.5%) | 116/432 (26.9%) | 95/345 (27.5%) | 61/205 (29.8%) | 90/290 (31.0%) |
| Winter | 314/1272 (24.7%) | 109/432 (25.2%) | 95/345 (27.5%) | 49/205 (23.9%) | 61/290 (21.0%) |
| Spring | 310/1272 (24.4%) | 110/432 (25.5%) | 76/345 (22.0%) | 43/205 (21.0%) | 81/290 (27.9%) |
| Antibiotics during labour (N=1253) | 277/1253 (22.1%) | 94/424 (22.2%) | 87/341 (25.5%) | 42/203 (20.7%) | 54/285 (18.9%) |
| Rupture of membrane > 24hrs (N=1233) | 91/1233 (7.4%) | 35/417 (8.4%) | 28/333 (8.4%) | 17/200 (8.5%) | 11/283 (3.9%) |
| Timing of randomisation (N=1269) |  |  |  |  |  |
| Aug 2013 - Feb 2015 | 474/1269 (37.4%) | 161/430 (37.4%) | 118/345 (34.2%) | 78/205 (38.0%) | 117/289 (40.5%) |
| March 2015 - Feb 2016 | 512/1269 (40.3%) | 176/430 (40.9%) | 136/345 (39.4%) | 81/205 (39.5%) | 119/289 (41.2%) |
| March 2016 - Sept 2016 | 283/1269 (22.3%) | 93/430 (21.6%) | 91/345 (26.4%) | 46/205 (22.4%) | 53/289 (18.3%) |
| Infant factors |  |  |  |  |  |
| Participant's sex (N=1272) |  |  |  |  |  |
| Female | 630/1272 (49.5%) | 226/432 (52.3%) | 171/345 (49.6%) | 92/205 (44.9%) | 141/290 (48.6%) |
| Male | 642/1272 (50.5%) | 206/432 (47.7%) | 174/345 (50.4%) | 113/205 (55.1%) | 149/290 (51.4%) |
| Gestational age at birth (decimal) (N=1272) | mean=39.3 (sd 1.4) | mean=39.4 (sd 1.4) | mean=39.3 (sd 1.3) | mean=39.4 (sd 1.4) | mean=39.1 (sd 1.6) |
| Preterm (N=1272) | 35/1272 (2.8%) | 11/432 (2.5%) | 6/345 (1.7%) | 5/205 (2.4%) | 13/290 (4.5%) |
| Birth weight, kg (N=1272) | mean=3.4 (sd 0.5) | mean=3.4 (sd 0.5) | mean=3.4 (sd 0.5) | mean=3.4 (sd 0.5) | mean=3.4 (sd 0.5) |
| Birth weight <2500 grams (N=1272) | 45/1272 (3.5%) | 11/432 (2.5%) | 13/345 (3.8%) | 5/205 (2.4%) | 16/290 (5.5%) |
| Antibiotics prior to discharge from birth hospital (N=1272) | 80/1272 (6.3%) | 23/432 (5.3%) | 21/345 (6.1%) | 10/205 (4.9%) | 26/290 (9.0%) |
| Hepatitis B vaccination up to 24 hours after randomisation (N=1271) | 1103/1271 (86.8%) | 364/432 (84.3%) | 302/345 (87.5%) | 177/204 (86.8%) | 260/290 (89.7%) |
| Birth order (N=1272) |  |  |  |  |  |
| 1st child | 686/1272 (53.9%) | 237/432 (54.9%) | 193/345 (55.9%) | 118/205 (57.6%) | 138/290 (47.6%) |
| 2^nd^ child | 396/1272 (31.1%) | 128/432 (29.6%) | 105/345 (30.4%) | 60/205 (29.3%) | 103/290 (35.5%) |
| 3rd child | 149/1272 (11.7%) | 56/432 (13.0%) | 40/345 (11.6%) | 17/205 (8.3%) | 36/290 (12.4%) |
| 4th child | 31/1272 (2.4%) | 8/432 (1.9%) | 4/345 (1.2%) | 9/205 (4.4%) | 10/290 (3.4%) |
| 5th child | 10/1272 (0.8%) | 3/432 (0.7%) | 3/345 (0.9%) | 1/205 (0.5%) | 3/290 (1.0%) |
| SCN/NICU admission >24 hours (prior to randomisation) (N=1268) | 94/1268 (7.4%) | 31/431 (7.2%) | 19/345 (5.5%) | 11/204 (5.4%) | 33/288 (11.5%) |
| Vitamin D supplementation in hospital (N=1268) | 370/1268 (29.2%) | 114/431 (26.5%) | 86/344 (25.0%) | 71/203 (35.0%) | 99/290 (34.1%) |
| Ancestry/ethnic origin of the child’s biological father’s mother (N=1190) |  |  |  |  |  |
| Aboriginal/Torres Strait | 11/1190 (0.9%) | 2/412 (0.5%) | 1/323 (0.3%) | 2/188 (1.1%) | 6/267 (2.2%) |
| African | 17/1190 (1.4%) | 3/412 (0.7%) | 4/323 (1.2%) | 4/188 (2.1%) | 6/267 (2.2%) |
| Asian | 119/1190 (10.0%) | 39/412 (9.5%) | 33/323 (10.2%) | 22/188 (11.7%) | 25/267 (9.4%) |
| British/Irish | 708/1190 (59.5%) | 250/412 (60.7%) | 193/323 (59.8%) | 108/188 (57.4%) | 157/267 (58.8%) |
| European | 274/1190 (23.0%) | 101/412 (24.5%) | 81/323 (25.1%) | 42/188 (22.3%) | 50/267 (18.7%) |
| Middle East | 36/1190 (3.0%) | 9/412 (2.2%) | 4/323 (1.2%) | 7/188 (3.7%) | 16/267 (6.0%) |
| Pacific Islander | 14/1190 (1.2%) | 3/412 (0.7%) | 3/323 (0.9%) | 3/188 (1.6%) | 5/267 (1.9%) |
| South American | 7/1190 (0.6%) | 4/412 (1.0%) | 1/323 (0.3%) | 0/188 (0.0%) | 2/267 (0.7%) |
| Other | 4/1190 (0.3%) | 1/412 (0.2%) | 3/323 (0.9%) | 0/188 (0.0%) | 0/267 (0.0%) |
| Ancestry/ethnic origin of child’s biological father’s father (N=1184) |  |  |  |  |  |
| Aboriginal/Torres Strait | 3/1184 (0.3%) | 0/408 (0.0%) | 0/314 (0.0%) | 2/191 (1.0%) | 1/271 (0.4%) |
| African | 19/1184 (1.6%) | 4/408 (1.0%) | 4/314 (1.3%) | 5/191 (2.6%) | 6/271 (2.2%) |
| Asian | 107/1184 (9.0%) | 38/408 (9.3%) | 29/314 (9.2%) | 19/191 (9.9%) | 21/271 (7.7%) |
| British/Irish | 702/1184 (59.3%) | 245/408 (60.0%) | 189/314 (60.2%) | 103/191 (53.9%) | 165/271 (60.9%) |
| European | 297/1184 (25.1%) | 105/408 (25.7%) | 85/314 (27.1%) | 53/191 (27.7%) | 54/271 (19.9%) |
| Middle East | 38/1184 (3.2%) | 11/408 (2.7%) | 4/314 (1.3%) | 7/191 (3.7%) | 16/271 (5.9%) |
| Pacific Islander | 10/1184 (0.8%) | 1/408 (0.2%) | 1/314 (0.3%) | 2/191 (1.0%) | 6/271 (2.2%) |
| South American | 7/1184 (0.6%) | 4/408 (1.0%) | 1/314 (0.3%) | 0/191 (0.0%) | 2/271 (0.7%) |
| Other | 1/1184 (0.1%) | 0/408 (0.0%) | 1/314 (0.3%) | 0/191 (0.0%) | 0/271 (0.0%) |
| Ancestry/ethnic origin of child’s biological mother’s father (N=1231) |  |  |  |  |  |
| Aboriginal/Torres Strait | 2/1231 (0.2%) | 0/415 (0.0%) | 1/335 (0.3%) | 0/197 (0.0%) | 1/284 (0.4%) |
| African | 17/1231 (1.4%) | 8/415 (1.9%) | 4/335 (1.2%) | 4/197 (2.0%) | 1/284 (0.4%) |
| Asian | 140/1231 (11.4%) | 43/415 (10.4%) | 40/335 (11.9%) | 28/197 (14.2%) | 29/284 (10.2%) |
| British/Irish | 757/1231 (61.5%) | 267/415 (64.3%) | 208/335 (62.1%) | 113/197 (57.4%) | 169/284 (59.5%) |
| European | 253/1231 (20.6%) | 80/415 (19.3%) | 73/335 (21.8%) | 40/197 (20.3%) | 60/284 (21.1%) |
| Middle East | 34/1231 (2.8%) | 8/415 (1.9%) | 6/335 (1.8%) | 8/197 (4.1%) | 12/284 (4.2%) |
| Pacific Islander | 11/1231 (0.9%) | 2/415 (0.5%) | 1/335 (0.3%) | 3/197 (1.5%) | 5/284 (1.8%) |
| South American | 13/1231 (1.1%) | 5/415 (1.2%) | 2/335 (0.6%) | 1/197 (0.5%) | 5/284 (1.8%) |
| Other | 4/1231 (0.3%) | 2/415 (0.5%) | 0/335 (0.0%) | 0/197 (0.0%) | 2/284 (0.7%) |
| Ancestry/ethnic origin of child’s biological mother’s mother (N=1243) |  |  |  |  |  |
| Aboriginal/Torres Strait | 7/1243 (0.6%) | 1/422 (0.2%) | 2/337 (0.6%) | 3/200 (1.5%) | 1/284 (0.4%) |
| African | 17/1243 (1.4%) | 8/422 (1.9%) | 1/337 (0.3%) | 5/200 (2.5%) | 3/284 (1.1%) |
| Asian | 153/1243 (12.3%) | 45/422 (10.7%) | 44/337 (13.1%) | 28/200 (14.0%) | 36/284 (12.7%) |
| British/Irish | 772/1243 (62.1%) | 276/422 (65.4%) | 209/337 (62.0%) | 116/200 (58.0%) | 171/284 (60.2%) |
| European | 236/1243 (19.0%) | 76/422 (18.0%) | 71/337 (21.1%) | 37/200 (18.5%) | 52/284 (18.3%) |
| Middle East | 32/1243 (2.6%) | 7/422 (1.7%) | 5/337 (1.5%) | 7/200 (3.5%) | 13/284 (4.6%) |
| Pacific Islander | 9/1243 (0.7%) | 2/422 (0.5%) | 1/337 (0.3%) | 3/200 (1.5%) | 3/284 (1.1%) |
| South American | 13/1243 (1.0%) | 6/422 (1.4%) | 3/337 (0.9%) | 1/200 (0.5%) | 3/284 (1.1%) |
| Other | 4/1243 (0.3%) | 1/422 (0.2%) | 1/337 (0.3%) | 0/200 (0.0%) | 2/284 (0.7%) |
| Plurality (N=1272) |  |  |  |  |  |
| Single pregnancy | 1251/1272 (98.3%) | 425/432 (98.4%) | 340/345 (98.6%) | 201/205 (98.0%) | 285/290 (98.3%) |
| Multiple pregnancy | 21/1272 (1.7%) | 7/432 (1.6%) | 5/345 (1.4%) | 4/205 (2.0%) | 5/290 (1.7%) |
| Familial or environmental factors |  |  |  |  |  |
| Any family member history of doctor diagnosed eczema (N=1270) | 514/1270 (40.5%) | 175/432 (40.5%) | 140/344 (40.7%) | 76/204 (37.3%) | 123/290 (42.4%) |
| Both parents have eczema (N=1270) | 31/1270 (2.4%) | 6/432 (1.4%) | 10/344 (2.9%) | 6/204 (2.9%) | 9/290 (3.1%) |
| Any family member history of food allergy (N=1252) | 195/1252 (15.6%) | 74/425 (17.4%) | 38/343 (11.1%) | 36/201 (17.9%) | 47/283 (16.6%) |
| Any family member history of doctor diagnosed asthma (N=1269) | 615/1269 (48.5%) | 217/431 (50.3%) | 154/345 (44.6%) | 100/204 (49.0%) | 144/289 (49.8%) |
| Either parent has a history of asthma (N=1269) | 577/1269 (45.5%) | 205/431 (47.6%) | 145/345 (42.0%) | 93/204 (45.6%) | 134/289 (46.4%) |
| Any family member history of hayfever (N=1270) | 836/1270 (65.8%) | 308/431 (71.5%) | 223/345 (64.6%) | 120/204 (58.8%) | 185/290 (63.8%) |
| Family history of ANY atopic disease - eczema, hayfever, asthma (N=1271) | 1049/1271 (82.5%) | 365/432 (84.5%) | 283/345 (82.0%) | 164/204 (80.4%) | 237/290 (81.7%) |
| Both parents have ANY atopic disease - eczema, hayfever, asthma (N=1269) | 386/1269 (30.4%) | 127/431 (29.5%) | 105/344 (30.5%) | 65/204 (31.9%) | 89/290 (30.7%) |
| Family history of ANY allergy -food, secondary, eczema, hayfever, asthma (N=1262) | 1070/1262 (84.8%) | 372/428 (86.9%) | 286/344 (83.1%) | 170/202 (84.2%) | 242/288 (84.0%) |
| Any family member has history of secondary allergy (N=1231) | 306/1231 (24.9%) | 96/420 (22.9%) | 85/335 (25.4%) | 57/197 (28.9%) | 68/279 (24.4%) |
| Family has a pet dog, birth timepoint (N=1272) | 503/1272 (39.5%) | 181/432 (41.9%) | 120/345 (34.8%) | 75/205 (36.6%) | 127/290 (43.8%) |
| Family has a pet cat, birth timepoint (N=1272) | 317/1272 (24.9%) | 117/432 (27.1%) | 79/345 (22.9%) | 44/205 (21.5%) | 77/290 (26.6%) |
| Family has livestock, birth timepoint (N=1271) | 48/1271 (3.8%) | 13/432 (3.0%) | 14/345 (4.1%) | 9/204 (4.4%) | 12/290 (4.1%) |
| Family owned an animal with hair at birth (N=1271) | 708/1271 (55.7%) | 256/432 (59.3%) | 176/345 (51.0%) | 103/204 (50.5%) | 173/290 (59.7%) |
| Smokers living in the house during pregnancy (N=1269) | 222/1269 (17.5%) | 59/432 (13.7%) | 43/345 (12.5%) | 46/204 (22.5%) | 74/288 (25.7%) |
| Number of household habitants (N=1272) | mean=2.9 (sd 1.1) | mean=2.8 (sd 1.1) | mean=2.8 (sd 1.0) | mean=3.0 (sd 1.3) | mean=3.0 (sd 1.1) |
| Number of household habitants (N=1272) |  |  |  |  |  |
| 1 | 16/1272 (1.3%) | 6/432 (1.4%) | 3/345 (0.9%) | 4/205 (2.0%) | 3/290 (1.0%) |
| 2 | 575/1272 (45.2%) | 198/432 (45.8%) | 168/345 (48.7%) | 93/205 (45.4%) | 116/290 (40.0%) |
| 3 | 391/1272 (30.7%) | 133/432 (30.8%) | 102/345 (29.6%) | 53/205 (25.9%) | 103/290 (35.5%) |
| 4 | 190/1272 (14.9%) | 65/432 (15.0%) | 54/345 (15.7%) | 31/205 (15.1%) | 40/290 (13.8%) |
| 5 | 62/1272 (4.9%) | 18/432 (4.2%) | 10/345 (2.9%) | 15/205 (7.3%) | 19/290 (6.6%) |
| 6 | 23/1272 (1.8%) | 5/432 (1.2%) | 6/345 (1.7%) | 6/205 (2.9%) | 6/290 (2.1%) |
| 7 | 11/1272 (0.9%) | 6/432 (1.4%) | 2/345 (0.6%) | 1/205 (0.5%) | 2/290 (0.7%) |
| 8 | 2/1272 (0.2%) | 1/432 (0.2%) | 0/345 (0.0%) | 0/205 (0.0%) | 1/290 (0.3%) |
| 9 | 1/1272 (0.1%) | 0/432 (0.0%) | 0/345 (0.0%) | 1/205 (0.5%) | 0/290 (0.0%) |
| 11 | 1/1272 (0.1%) | 0/432 (0.0%) | 0/345 (0.0%) | 1/205 (0.5%) | 0/290 (0.0%) |
| Number of household habitants under school age (N=1271) |  |  |  |  |  |
| 0 | 756/1271 (59.5%) | 251/432 (58.1%) | 221/345 (64.1%) | 133/204 (65.2%) | 151/290 (52.1%) |
| 1 | 417/1271 (32.8%) | 144/432 (33.3%) | 101/345 (29.3%) | 57/204 (27.9%) | 115/290 (39.7%) |
| 2 | 90/1271 (7.1%) | 34/432 (7.9%) | 22/345 (6.4%) | 13/204 (6.4%) | 21/290 (7.2%) |
| 3 | 7/1271 (0.6%) | 3/432 (0.7%) | 1/345 (0.3%) | 1/204 (0.5%) | 2/290 (0.7%) |
| 4 | 1/1271 (0.1%) | 0/432 (0.0%) | 0/345 (0.0%) | 0/204 (0.0%) | 1/290 (0.3%) |
| Sibling/household under school age attending non-parent care anytime (N=1155) | 349/1155 (30.2%) | 124/404 (30.7%) | 93/317 (29.3%) | 49/175 (28.0%) | 83/259 (32.0%) |
| Environmental factors at 1 year |  |  |  |  |  |
| Family has a pet dog, at 1 year (N=1147) | 434/1147 (37.8%) | 175/424 (41.3%) | 115/343 (33.5%) | 52/158 (32.9%) | 92/222 (41.4%) |
| Family has a pet cat, at 1 year (N=1147) | 265/1147 (23.1%) | 101/424 (23.8%) | 75/343 (21.9%) | 29/158 (18.4%) | 60/222 (27.0%) |
| Sibling/household under school age children attending non-parent care anytime at 1 year (N=1155) | 349/1155 (30.2%) | 124/404 (30.7%) | 93/317 (29.3%) | 49/175 (28.0%) | 83/259 (32.0%) |
| Daycare attendance for participant during first year of life (N=1242) | 457/1242 (36.8%) | 168/432 (38.9%) | 144/345 (41.7%) | 51/196 (26.0%) | 94/269 (34.9%) |
| Participant or sibling attended childcare during participants first year of life (N=1241) | 691/1241 (55.7%) | 253/429 (59.0%) | 198/344 (57.6%) | 95/194 (49.0%) | 145/274 (52.9%) |
| Any travel overseas in first year of life (N=1148) | 257/1148 (22.4%) | 100/424 (23.6%) | 74/344 (21.5%) | 40/159 (25.2%) | 43/221 (19.5%) |
| Age at first DTP vaccination (usually 6w infanrix) (N=1240) | mean=50.0 (sd 34.4) | mean=48.9 (sd 12.6) | mean=48.1 (sd 8.6) | mean=51.0 (sd 21.4) | mean=53.3 (sd 68.7) |
| Participant hospitalised for bronchiolitis during the first year of life (N=1054) | 33/1054 (3.1%) | 15/407 (3.7%) | 6/320 (1.9%) | 4/138 (2.9%) | 8/189 (4.2%) |
| Participant atopic in first year of life (eczema or SPT pos) (N=1043) | 470/1043 (45.1%) | 158/406 (38.9%) | 144/325 (44.3%) | 78/134 (58.2%) | 90/178 (50.6%) |
| Environmental factors at 5 years |  |  |  |  |  |
| Family had a pet cat, during child's first 5 years of life (N=1070) | 396/1070 (37.0%) | 148/430 (34.4%) | 114/341 (33.4%) | 53/122 (43.4%) | 81/177 (45.8%) |
| Family has a pet cat, at 5 years (N=1003) | 182/1003 (18.1%) | 102/430 (23.7%) | 74/340 (21.8%) | 3/93 (3.2%) | 3/140 (2.1%) |
| Family had a pet dog, during child's first 5 years of life (N=1110) | 583/1110 (52.5%) | 216/430 (50.2%) | 151/343 (44.0%) | 78/132 (59.1%) | 138/205 (67.3%) |
| Family has a pet dog, at 5 years (N=1003) | 264/1003 (26.3%) | 152/430 (35.3%) | 103/340 (30.3%) | 3/93 (3.2%) | 6/140 (4.3%) |
| Family had livestock, during child's first 5 years of life (N=741) | 103/741 (13.9%) | 34/388 (8.8%) | 35/302 (11.6%) | 14/19 (73.7%) | 20/32 (62.5%) |
| Any evidence of smoke exposure during the child's first 5 years of life (N=1272) | 279/1272 (21.9%) | 85/432 (19.7%) | 63/345 (18.3%) | 53/205 (25.9%) | 78/290 (26.9%) |
| Breastfeeding cessation by (N=1177) |  |  |  |  |  |
| no breastfeeding | 29/1177 (2.5%) | 6/431 (1.4%) | 4/345 (1.2%) | 7/172 (4.1%) | 12/229 (5.2%) |
| <1 week | 33/1177 (2.8%) | 7/431 (1.6%) | 7/345 (2.0%) | 10/172 (5.8%) | 9/229 (3.9%) |
| 1wk - <=3 months | 207/1177 (17.6%) | 70/431 (16.2%) | 41/345 (11.9%) | 37/172 (21.5%) | 59/229 (25.8%) |
| >3 - 6 months | 127/1177 (10.8%) | 37/431 (8.6%) | 38/345 (11.0%) | 22/172 (12.8%) | 30/229 (13.1%) |
| >6 - 9 months | 126/1177 (10.7%) | 46/431 (10.7%) | 41/345 (11.9%) | 20/172 (11.6%) | 19/229 (8.3%) |
| >9 - 12 months | 168/1177 (14.3%) | 68/431 (15.8%) | 52/345 (15.1%) | 15/172 (8.7%) | 33/229 (14.4%) |
| >12 - 18 months | 278/1177 (23.6%) | 103/431 (23.9%) | 85/345 (24.6%) | 36/172 (20.9%) | 54/229 (23.6%) |
| >18 - 24 months | 104/1177 (8.8%) | 52/431 (12.1%) | 34/345 (9.9%) | 12/172 (7.0%) | 6/229 (2.6%) |
| >24-36 months | 73/1177 (6.2%) | 31/431 (7.2%) | 28/345 (8.1%) | 9/172 (5.2%) | 5/229 (2.2%) |
| >36 months | 32/1177 (2.7%) | 11/431 (2.6%) | 15/345 (4.3%) | 4/172 (2.3%) | 2/229 (0.9%) |

**Supplementary Table E2: Subgroup analysis of incidence of asthma at 5 years of age in children with or without allergic sensitisation**

|  | BCG | Control |
| --- | --- | --- |
|  | N=637 | N=635 |
| No asthma and no allergic sensitisation | 307 (48.2%) | 236 (37.2%) |
| No asthma but presence of allergic sensitisation | 73 (11.5%) | 58 (9.1%) |
| Asthma but no allergic sensitisation | 32 (5.0%) | 21 (3.3%) |
| Asthma and allergic sensitisation (‘atopic asthma’) | 10 (1.6%) | 16 (2.5%) |
| Missing data on asthma and/or allergic sensitisation | 215 (33.8%) | 304 (47.9%) |

**Supplementary Figure E1: Subgroup analysis of incidence of asthma at 5 years of age in children with or without allergic sensitisation**


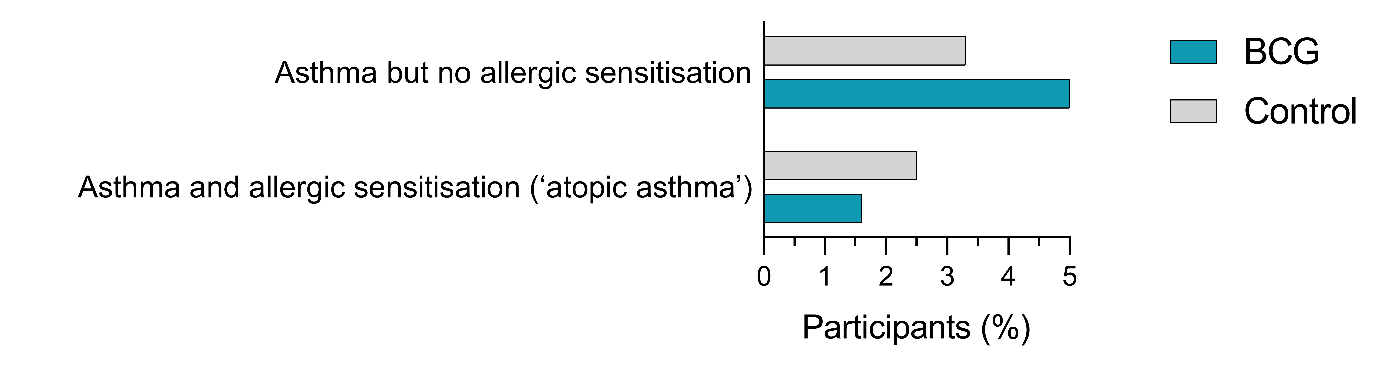


**Sensitivity analysis and complete case analysis**

|  | BCG | Control | Adjusted risk difference (95% CI)* |
| --- | --- | --- | --- |
| SENSITIVITY ANALYSIS (multiple imputation) | | | |
| Primary outcome - asthma ever at 5 years of age **without** catchup questionnaire data | 14.7% | 16.2% | -1.6 (-7.5, 4.3) |

* Adjusted for stratification variable: mode of delivery

|  | BCG | Control | Adjusted risk difference (95% CI)* |
| --- | --- | --- | --- |
| COMPLETE CASE ANALYSIS | | | |
| Primary outcome |  |  |  |
| Asthma at 5 years of age | 44/432 (10.2%) | 40/345 (11.6%) | -1.8 (-6.2, 2.6) |
| Secondary outcomes |  |  |  |
| Current asthma at 5 years of age | 32/437 (7.3%) | 29/351 (8.3%) | -1.5 (-5.3, 2.2) |
| Wheeze within the last 12 months at 5 years of age | 55/388 (14.2%) | 51/306 (16.7%) | -2.7 (-8.1, 2.7) |
| Severe asthma at 5 years of age | 16/388 (4.1%) | 21/306 (6.9%) | -2.5 (-5.9, 0.8) |
| Preventer medication in the last 12 months at 5 years of age | 17/385 (4.4%) | 22/305 (7.2%) | -2.9 (-6.4, 0.7) |

* Adjusted for stratification variable: mode of delivery

**Multiple imputation**

Overall, the proportion of children with missing data for the primary and secondary outcomes ranged from 38.9% to 45.6%. Multiple imputation (MI) by chained equations was conducted to handle missing data, as this approach to MI can handle multivariable missingness. A single imputation model with all outcome variables included would not converge therefore, an individual imputation model was estimated for the primary outcome and secondary outcomes of current asthma and use of preventer medication. The secondary outcome of wheeze disorder and severity were estimated in a single conditional imputation model because the severity variable was conditional on wheeze being present. The table below shows the variables identified a priori that were planned to be included in the MI and indicates if that variable was complete (or not) and if they were able to be included in the imputation models.

| **Variables in model** |  | Primary and secondary outcomes imputation models (X indicates variable was included) | | | | |
| --- | --- | --- | --- | --- | --- | --- |
|  | Missingness for each variable | incidence of asthma (primary outcome) | current (active) asthma (secondary outcome) | use of preventer medication (secondary outcome) | Conditional imputation: current “wheezing disorder” (secondary outcome) | Conditional imputation: asthma severity (secondary outcome,) |
| Auxiliary variables |  |  |  |  |  |  |
| Maternal age at delivery | 1 (0.1%) | X | X | X |  |  |
| Mother education level categorized (3 categories) | 3 (0.2%) | X | X | X |  |  |
| Paternal age at delivery | 33 (2.6%) | X | X | X |  |  |
| Season of Birth | 0 | X | X | X | X | X |
| Timing (3 categories for pre/post covid and post survey update) | 3 (0.2%) | X | X | X |  |  |
| Infant birthweight <2500 grams | 0 | X | X | X | X | X |
| Participant's sex | 0 | X | X | X | X | X |
| Family owned a pet with hair at birth | 1 (0.1%) | X | X | X | X | X |
| Any family member history of doctor diagnosed asthma (mother, father, siblings) | 3 (0.2%) | X | X | X | X | X |
| Sibling or participant attended childcare during participant's first year of life | 31 (2.4%) | X | X | X |  |  |
| Hospitalisation for bronchiolitis | 218 (17.1%) | X | X | X |  |  |
| Participant had atopic disease during first year | 229 (18.0%) | X | X | X |  |  |
| Smokers living in the house (ever) | 0 | X | X | X | X | X |
| Cease breastfeeding updated for part 2 (5 categories) | 95 (7.5%) | X | X | X |  |  |
| Evidence of asthma | 414 (32.5%) | X | X | X | X | X |
| Stratum variable |  |  |  |  |  |  |
| Mode of delivery (binary) | 0 | X | X | X | X | X |
| Outcome variables |  |  |  |  |  |  |
| incidence of asthma (primary outcome) | 495 (38.9%) | X |  |  |  |  |
| current (active) asthma (secondary outcome) | 484 (38.1%) |  | X |  |  |  |
| use of preventer medication (secondary outcome) | 582 (45.8%) |  |  | X |  |  |
| Current “wheezing disease” (secondary outcome) | 578 (45.4%) |  |  |  | X | X |
| Asthma severity (secondary outcome, need to be positive to wheezing) | 578 (45.4%) |  |  |  | X | X |
| Subgroup variables |  |  |  |  |  |  |
| Parent asthma | 3 (0.2%) |  |  |  |  |  |
| Sex of infant | 0 |  |  |  |  |  |
| Maternal BCG | 66 (5.2%) |  |  |  |  |  |
| SPT pos (≥2mm >neg control) to at least one allergen, test done at 11-24months | 206 (16.2%) |  |  |  |  |  |
