# Supplementary material for: Neonatal BCG vaccination to prevent asthma: Results from the MIS BAIR randomized controlled trial
Source: Pediatr Allergy Immunol. 2025 Jun 4;36(6):e70110. doi: 10.1111/pai.70110 (PMC12136015; doi:10.1111/pai.70110)
Supplement: Supplementary file 2 — Data S2. Supporting Information. [file PAI-36-e70110-s002.pdf]

FORM-STAT-04A-01	Statistical Analysis Plan
Version 2.0	

BCG12/01

The Melbourne Infant Study
BCG for Allergy & Infection Reduction
(MIS BAIR)
Asthma outcome

Document Version History

Version Date	Version	Author	Signature	Change Description	Reason/Comment
December 2023	1	Emily Forbes		Initial release.	Not applicable.
15 January 2024	2	Emily Forbes		Secondary outcomes edited. Sensitivity analysis added	Follow-up questionnaire removed from some outcomes and sensitivity analysis added for primary outcome

TABLE OF CONTENTS

LIST OF ABBREVIATIONS **3**

1. STUDY OBJECTIVES **4**
- 1.1. PRIMARY OBJECTIVE 4
- 1.2. SECONDARY OBJECTIVES 4

2. BACKGROUND/INTRODUCTION **4**
- 2.1. STUDY DESIGN 4
- 2.2. INTERVENTION GROUPS 4
- 2.3. STUDY POPULATION 5
- 2.4. SAMPLE SIZE 6
- 2.5. STUDY PROCEDURE 7

3. POPULATIONS OF ANALYSIS **7**

4. OUTCOME VARIABLES **7**
- 4.1. DATA COLLECTION 7
- 4.2. PRIMARY OUTCOMES 9
- 4.3. SECONDARY OUTCOMES 10
- 4.4. OTHER PARAMETERS .... **ERROR! BOOKMARK NOT DEFINED.**

5. STATISTICAL METHODOLOGY **11**
- 5.1. GENERAL METHODOLOGY 11
- 5.2. DESCRIPTIVE STATISTICS 12
- 5.3. CLASSIFICATION OF PROTOCOL VIOLATION .... **ERROR! BOOKMARK NOT DEFINED.**
- 5.4. PRIMARY DATA ANALYSES 15
- 5.5. SECONDARY DATA ANALYSES 15
- 5.6. ANALYSIS OF OTHER OUTCOMES 15

6. REFERENCES **16**

LIST OF ABBREVIATIONS

AE	Adverse Event
BCG	Bacille Calmette-Guérin
CRF	Case Report Form
DAG	Directed Acyclic Graph
GCP	Good Clinical Practice
ISAAC	International Study of Asthma and Allergies in Childhood
ITT	Intent-To-Treat
LAR	Legally acceptable representative
MIS BAIR	The Melbourne Infant Study: BCG for Allergy & Infection Reduction
PI	Principal Investigator
PICF	Participant information and consent form
SAE	Serious Adverse Event
SAP	Statistical Analysis Plan
SD	Standard Deviation
SE	Standard Error

1. STUDY OBJECTIVES

1.1. PRIMARY OBJECTIVE

To determine if in healthy neonates (Participants), BCG vaccination in the first ten days of life (Intervention), compared to no BCG vaccination (Comparator), reduces the incidence of 'asthma ever' (Outcome) at five years of age (Time).

1.2. SECONDARY OBJECTIVES

To determine if in healthy neonates (Participants), BCG vaccination at birth (Intervention), compared to no BCG vaccination (Comparator) reduces the:

1. incidence of current asthma (Outcome) at five years of age (Time)
2. incidence of current wheeze (Outcome) at five years of age (Time)
3. incidence of severe asthma (Outcome) at five years of age (Time)
4. use of preventer and reliever asthma medication (Outcome) at five years of age (Time)

Note that the MIS BAIR study has other outcomes for allergy, infection and eczema at both one and five years of age. This Statistical Analysis Plan relates only to the asthma outcomes.

2. BACKGROUND/INTRODUCTION

2.1. STUDY DESIGN

MIS BAIR is a phase III, randomised, controlled trial in infants to determine if the bacille Calmette-Guérin (BCG) vaccine reduces the incidence of eczema, allergies, infections and asthma in the first five years of life.

Infants were recruited at Mercy Hospital for Women, Werribee Mercy, Geelong Hospital, St John of God and Murdoch Children's Research Institute in Melbourne over a three-year period from August 2013 to September 2016. At trial design we planned to randomise 1,438 infants 1:1 to receive the BCG vaccine or no vaccine. However, recruitment to the study was stopped before this number was reached as the BCG-Denmark vaccine became unavailable due to a worldwide shortage. The actual number randomised was 1272. Infants were randomised within ten days of birth.

The study involved two parts:

Part 1 – From birth to one year of age
Part 2 – From one to five years of age

At the 1-year MIS BAIR visit participants were asked to re-consent if they wished to continue into Part 2 of the study. For those families who had not been consented at the 1-year MIS BAIR visit, had not verbally declined participation and had given consent to be contacted for future studies, an invitation letter and the PICF were sent to them by email, post or through REDCap and they could consent by post, on REDCap or in person (e.g. at a study visit). Of the 1272 in Part 1, 1027 consented to continue into Part 2.

2.2. INTERVENTION GROUPS

Randomisation was stratified by:

(i) site (hospital),
(ii) method of delivery (Caesarean vs non-Caesarean) and
(iii) plurality of birth (i.e. twins vs singletons).

Neonates were randomised within 10 days of birth to BCG vaccine or no BCG vaccine. Those randomised to BCG vaccine were to receive the vaccine within 24hrs of randomisation (and still within 10 days of birth).

Study participants randomised to group 1 received a single 0.05 mL intradermal dose of BCG-Denmark vaccine SSI (a freeze-dried live vaccine prepared from an attenuated strain of *Mycobacterium bovis*) over the left deltoid, within 10 days of birth. Vaccination could be deferred where the infant was medically unstable, until the infant was in good medical condition as determined by the treating clinician, for a maximum period of 24 hours.

A total of 57 participants were vaccinated with BCG vaccine batch 114010C (expiry 06/2016) after the manufacture assigned used-by-date within three months of expiration (use past the expiry date for the study was approved by the governing HRECs).

At the 1-year and 5-year visits, the member of the research team doing the assessment was blinded to treatment group allocation - they had no access to the randomisation allocation data and a bandage was applied to the upper left infant’s arm to hide any potential scar. Parents/guardians of participants could not be blinded to the treatment group allocation due to the localized reaction and scar formation following BCG vaccination.

2.3. STUDY POPULATION

1,272 healthy newborns were randomised, within 10 days of birth, in a 1:1 ratio, to study group 1 (BCG, n=637) or study group 2 (no BCG, n=635). Twin pairs were randomised to the same group but only one twin from each pair was included as a study participant, regardless of birth order. A total of 21 sets of twins were randomised in the trial and 17 sets of twins continued into Part 2.

2.3.1. Inclusion Criteria

For MIS BAIR (Part 1):

- Less than 10 days old;
- English speaking mother;
- An informed consent form must be signed and dated by their parent(s) or legally acceptable representative (LAR) after the nature of the study has been explained and prior to any study assessments/procedures;
- The infant’s mother has screened negative for HIV during this pregnancy;
    - To ensure this is accurate, before randomisation staff will check the mother’s pathology results to confirm the date of her HIV test is post conception for the current pregnancy (this date is recorded on the eligibly check form)
- Born no earlier than eight weeks before estimated date of delivery;
- Birth weight >1500g;
- The LAR expects to be able to complete four online/phone questionnaires over the infant’s first 12 months of life and for the infant to be available for skin prick testing at a study site at 1-year of age.

Additional inclusion criteria for part 2:

- Were randomised in part 1 of MIS BAIR

2.3.2. Exclusion Criteria

For MIS BAIR (Part 1):

An infant meeting any of the following criteria will be excluded from study participation (as per the NH&MRC Immunisation Handbook (10th edition, 2013)):

- Any indication for BCG immunisation in the first 12 months of life including:
  - likely travel to a high TB incidence country in the first year of life;
  - aboriginal and Torres Strait Islander babies living in parts of Australia where the incidence of TB is higher;
  - newborn babies, if either parent has leprosy or a family history of leprosy
  - newborn in contact with a patient with TB.
- Known or suspected HIV infection;
- Treatment with corticosteroids or other immunosuppressive therapy, including monoclonal antibodies against tumour necrosis factor-alpha (TNF-alpha) (e.g. infliximab, etanercept, adalimulab);
- Born to a mother treated with bDMARDS (e.g. TNF-alpha blocking monoclonal antibodies) in the 3rd trimester;
- Congenital cellular immunodeficiencies including specific deficiencies of the interferon gamma pathway;
- Malignancies involving bone marrow or lymphoid systems;
- Serious underlying illness including severe malnutrition;
- Medically unstable;
- Generalised septic skin disease and skin conditions such as eczema, dermatitis and psoriasis;
- Significant febrile illness;
- Sibling in the study (other than a twin)
- Multiple births more than twins.
- Born to mother who has tested Hep B antigen positive in current pregnancy and whose baby is likely to be given hepatitis B immunoglobulin.
- Mother has received celestone/betamethasone 48 hours before randomisation

Also excluded are infants with:

- a mother who is immunosuppressed;
- a family history of immunodeficiency;
- consanguineous parents.

Additional exclusion criteria for part 2:

- Excluded during Part 1 of the study
- Withdrawn during Part 1 of the study

2.4. SAMPLE SIZE

The original sample size estimate (n=1438) was calculated for outcomes at the 1-year timepoint, rather than for the asthma outcomes. The sample size was calculated based on a 'worse case scenario' assumption that complete 1-year data would only be available for 80% of participants, i.e. 1,150. This sample size was powered to detect a 35% or greater reduction in skin prick test

positivity (i.e. from 18% to 12%), a 25% or greater reduction in eczema (i.e. from 27% to 20%), and a 25% or greater reduction in lower respiratory tract infection (i.e. from 27% to 20%) at 12 months of age[1].

The Asthma in Australia 2011 report[2], indicated that for children 4-5 years of age 15.4% have ‘current asthma’ defined as 'ever told by a doctor they have asthma’ AND 'yes' to 'In the last 12 months, has the child had an illness with wheezing in the chest which lasted for a week or more or taken any medication for asthma?' and 22% have ‘asthma ever’ defined as ‘ever been told by doctor that their child has asthma’. Assuming that 90% of participants would remain in the study to 5 years (n=1294), it was estimated that this sample size would allow detection of an absolute reduction in current asthma of 5.5% (15% to 9.5%) and an absolute reduction in ‘asthma ever’ of 6% (22% to 16%)[1].

The actual sample size for Part 1 was n=1272, due to the worldwide shortage of BCG vaccine. After the 1-year visit, participants were asked to consent to Part 2 of the study and 1027 families consented (80.7%).

2.5. STUDY PROCEDURE

The study design as proposed in the protocol is outlined below.

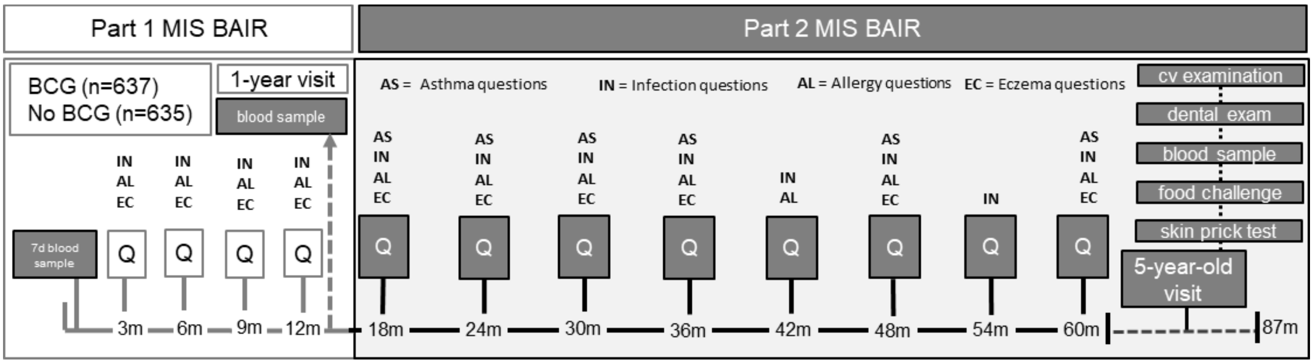

3. POPULATIONS OF ANALYSIS

An intention-to-treat (ITT) analysis will be used to analyse the outcomes, with all participants analysed according to the study group to which they were randomised, regardless of the intervention they actually received. The only participants excluded from analysis will be participants who withdrew consent for their data to be used.

Participants who did not consent to Part 2 will be treated as missing for any data that was collected during Part 2. They will continue to contribute data to baseline, 1-year outcomes and outcomes that depend on an event ‘ever’ happening.

4. OUTCOME VARIABLES

4.1. Data collection

4.1.1. Questionnaires

Parents/guardians of participants were asked to complete 3-monthly questionnaires to one year of age and then 6 and/or 12 monthly questionnaires from one year to five years of age.

These questionnaires collected data relevant to all the outcomes of the study including skin condition, reactions to allergens, episodes of illness, wheeze/asthma, and vaccinations. This SAP only outlines data collected that is relevant to the asthma outcomes.

4.1.2. ISAAC questions at five years of age

At five years of age the questionnaires included validated questions from the International Study of Asthma and Allergies in Childhood (ISAAC)[3].

These questions are:

1. *Has your child ever had wheezing or whistling in the chest at any time in the past?*
   Yes/No/Don't know

If they answer yes to the first question, the following question was asked:

2. Has your child had wheezing or whistling in the chest in the past 12 months?
Yes/No/Don't know

If they answer yes to this question, the following questions were asked:

3. How many attacks of wheezing has your child had in the past 12 months?
None | 1 to 3 | 4 to 12 | More than 12

4. In the past 12 months, how often, on average, has your child's sleep been disturbed due to wheezing?
Never woken with wheezing | Less than one night per week | One or more nights per week

5. In the past 12 months, has wheezing ever been severe enough to limit your child's speech to only one or two words at a time between breaths?
Yes/No

All participants were asked:

6. Has your child ever had asthma?
Yes/No/Don't know

7. In the past 12 months, has your child's chest sounded wheezy during or after exercise?
Yes/No

8. In the past 12 months, has your child had a dry cough at night, apart from a cough associated with a cold or chest infection?
Yes/No

4.1.3. Follow-up questionnaire for missing data

At seven to ten years of age parents/guardians of participants were asked to complete a follow-up questionnaire. The purpose of the questionnaire was to identify participants who had *not* had the outcome of interest. The questionnaire was only sent to participants who were known to have

missing data due to not completing all the questionnaires. Only 'no' responses will be taken as valid data.

For asthma, the question asked of participants was:

Has [child's name] ever had asthma? Yes/No/Unsure

4.1.4. Asthma medication

Preventer and reliever medication are commonly prescribed to children for wheeze and asthma. Whereas relievers are prescribed in acute phases, to relieve acute symptoms, preventers are prescribed for more severe asthma, to decrease inflammation and prevent episodes[4].

In the five-year questionnaire, if the parent/guardian indicated that the child had either asthma or wheezing, the parent/guardian was asked whether the child had ever used any medication, pills, puffers or other medicines for wheezing or asthma. If yes, they were presented a list of medications that included:

<u>Relievers</u>
Salbutamol (e.g Ventolin, Asmol)
Pulmicort (Budesonide)
Prednisolone (e.g Redipred, PredMix, Predsol)
Atrovent (Ipratropium Bromide)

<u>Preventers</u>
Flixotide (Fluticasone Propionate)
Seretide (e.g Fluticasone and Salmeterol)
Alvesco (Ciclesonide)
Singulair (Montelukast)
Symbicort (Formoterol)

If they selected any of the medications, they were then asked if the child had used it in the last 12 months.

Any 'other' medications listed will be classified as a preventer or reliever as appropriate.

4.2. PRIMARY OUTCOME

1. **Asthma ever, defined by ISAAC questions**
   Positive if the parent/guardian answered 'yes' to 'Has your child ever had asthma?' (ISAAC Q6).
   Negative if the parent/guardian answered
   - 'no' to 'Has your child ever had asthma' in the 5-year questionnaire (ISAAC Q6).
   OR
   - 'no' to the asthma question in the 7 – 10-year follow-up questionnaire

Missing if in the 5-year questionnaire the response to ISAAC Q6 is missing or don't know AND the response to the follow-up questionnaire is not 'no'.

4.3. SECONDARY OUTCOMES

1. **Current asthma at five years of age, defined by ISAAC questions**

   Positive if in the 5-year questionnaire the parent/guardian answered 'yes' to 'Has your child ever had wheezing or whistling in the chest in the chest at any time in the past?' (ISAAC Q1) AND 'Has your child had wheezing or whistling in the chest in the past 12 months?' (ISAAC Q2) AND 'Has your child ever had asthma?' (ISAAC Q6).

   Negative if in the 5-year questionnaire the parent/guardian answered:
   - 'no' to 'Has your child ever had wheezing or whistling in the chest in the chest at any time in the past?' (ISAAC Q1)

   OR
   - 'yes' to 'Has your child ever had wheezing or whistling in the chest in the chest at any time in the past?' (ISAAC Q1) AND 'no' to either or both of:
     - 'Has your child had wheezing or whistling in the chest in the past 12 months?' (ISAAC Q2)
     - 'Has your child ever had asthma?' (ISAAC Q6) in the five-year questionnaire.

     OR
   - 'no' to the asthma question in the follow-up questionnaire

   Missing if in the 5-year questionnaire responses to the above questions are missing AND the response the follow-up questionnaire is not 'no'
   OR
   The participant does not fulfil the 'positive' or 'negative' criteria above.

2. **Current wheeze at five years of age, defined by ISAAC questions**

   Positive if in the 5-year questionnaire the parent/guardian answered 'yes' to both 'Has your child ever had wheezing or whistling in the chest in the chest at any time in the past?' (ISAAC Q1) AND 'Has your child had wheezing or whistling in the chest in the past 12 months?' (ISAAC Q2).

   Negative if in the 5-year questionnaire the parent/guardian answered:
   - 'no' to 'Has your child ever had wheezing or whistling in the chest in the chest at any time in the past?' (ISAAC Q1)

   OR
   - yes to 'Has your child ever had wheezing or whistling in the chest in the chest at any time in the past?' (ISAAC Q1) AND 'no' to 'Has your child had wheezing or whistling in the chest in the past 12 months?' (ISAAC Q2).

   Missing if in the 5-year questionnaire responses to the ISAAC Q1, Q2 or Q6 is missing
   OR
   The participant does not fulfil the 'positive' or 'negative' criteria above.

3. **Asthma severity, defined by ISAAC questions**

Positive (severe asthma) if the child is positive for the outcome 'current wheeze at five years of age'', AND in the 5-year questionnaire the parent/guardian indicated that in the last 12 months the child had:

- 4 or more attacks of wheezing (ISAAC Q3)

OR

- disturbed sleep one or more nights per week (ISAAC Q4),

OR

- speech limited to one or two words by wheeze (ISAAC Q5).

Negative (mild or no asthma) if the child is:

- negative for the outcome 'current wheeze',

OR

- positive for the outcome 'current wheeze at five years of age' AND in the 5-year questionnaire the parent/guardian indicated that in the last 12 months the child had
  - 0 – 3 attacks of wheeze (ISAAC Q3),

  AND
  - disturbed sleep less than once per week (ISAAC Q4)

  AND
  - no speech limited by wheeze (ISAAC Q5).

Missing if the child is missing for the outcome 'current wheeze at five years of age' or ISAAC Q3,4 or 5 and not positive for any other
OR
The participant does not fulfil the 'positive' or 'negative' criteria above.

4. **Asthma severity, defined as use of asthma preventer medication at five years of age**

Positive (Severe asthma) if in the 5-year questionnaire the parent/guardian reports that the child has used a preventer medication in the last 12 months (with or without use of a reliever medication).
Negative (mild or no asthma) if in the 5-year questionnaire

- the parent/guardian reports that the child has not used any preventer medication in the last 12 months

OR

- responded 'no' to ISAAC Q1 and ISAAC Q6.

Missing if the asthma medication section of the 5-year questionnaire was not completed
OR
The participant does not fulfil the 'positive' or 'negative' criteria above.

5. STATISTICAL METHODOLOGY

5.1. GENERAL METHODOLOGY

Although the details of the randomisation groups have already been unblinded (to the trial statisticians and specific data managers/trial co-ordinators) and the results published for Part 1 of the study, the PIs, as well as individuals responsible for cleaning and preparing the data for Part 2 have remained blinded. The data will only be fully unblinded once the database has been locked and all the SAPs have been finalised, approved by the PIs and made publicly available.

The primary outcome will be considered in evaluating the effectiveness of the trial intervention at reducing incidence of asthma. The magnitude of the treatment effect, with 95% confidence intervals and p-value, will be estimated for the outcome.

5.2. DESCRIPTIVE STATISTICS

5.2.1. PARTICIPANT DISPOSITION

All participants who were invited to participate in MIS BAIR will be accounted for in the CONSORT diagram. The CONSORT diagram for Part 1 has already been published [5,6], and will be updated to include Part 2. The number and proportion of participants who did not consent to Part 2 and withdrew from the study will be presented by intervention group (BCG or no BCG). The number and proportion of respondents to the asthma section of the five-year questionnaire will also be presented by intervention group.

5.2.2. PARTICIPANT CHARACTERISTICS

The demographic characteristics of the participants (including infant, maternal, paternal and environmental factors) at randomisation in the ITT population will be presented for each intervention group (BCG or no BCG) using the mean and standard deviation (SD) or median and interquartile range (IQR) for continuous and using numbers and proportions for categorical data. Environmental factors that may change over time may also be presented at one year and five years of age and 'ever' exposed (Any exposure between zero to five years). The full list of demographic characteristics that may be included is shown in *Appendix 1*. Given most of these data are already published [5,6], discretion will be used in determining which variables to include in publications.

Participant characteristics at baseline, one year and five years for the subset of participants who consented to Part 2, and for the subset of participants who answered the asthma questions in the 5-year questionnaire, will be also be presented.

5.2.3. HANDLING OF MISSING DATA

Missing data for environmental exposures

To determine whether a participant has 'ever' been exposed to an environmental exposure such as smoking or pets, missing values will be treated as '0' (no), provided that the participant responded 'no' in any completed questionnaires and that the baseline and five-year questionnaires are complete. This relies on the assumption that it is unlikely that a household member will become a smoker and then stop again, and similarly it is unlikely that the household will acquire a dog or cat for a short period of time.

Multiple Imputation

The primary analysis will be an intention-to-treat analysis. It is already known that the proportion of missing data for the primary outcome will be more than 5%, due to loss to follow-up. To adjust for this selection bias (collider stratification bias), multiple imputation models will be conducted for the outcome variables and 50 completed data sets will be imputed by chained equations including all the children initially randomised. The primary outcome, strata variable (mode of birth: vaginal/caesarean), and the variables associated with either remaining in the study or asthma listed below will be included in the imputation model. Although there are two other randomisation strata (study site and singleton/twin), the number of participants recruited at study sites other than the Mercy, and the number of twins, is so small that it is not possible to multiply impute separately for these strata.

The directed acyclic graph (DAG) below summarizes the expected associations between variables, including the open front door path created by restriction on the collider ‘remain in the study’ caused by loss to follow-up and incomplete data. The multiple imputation aims to impute the missing data for those who did not remain in the study. By including variables that are expected to be associated with both remaining in the study and the outcome, the restriction on ‘remain in the study’ is removed and this open path is closed.

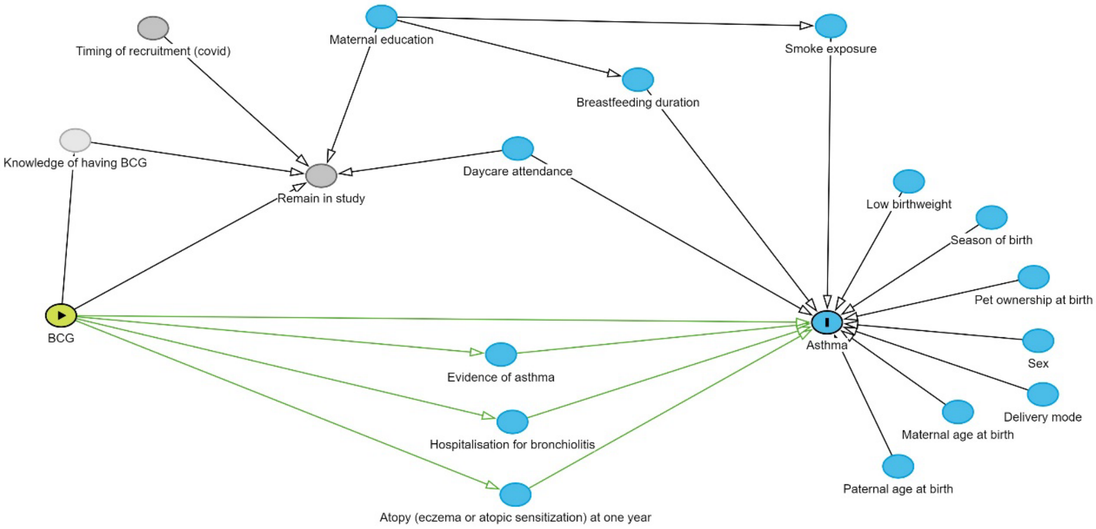

Informed by the DAG, and in order of priority, the variables listed below may be included in the multiple imputation. If necessary, the number of categories included in categorial variables will be reduced to allow the multiple imputation to run.

**Variable**		**Definition**
Maternal education	Generated, categorical	Three categories – below year 10, year 12/trade certificate or university educated.
Daycare attendance[7]	Generated, binary	Positive if the participant had a sibling in daycare at birth or during the first year of life, or if the participant attended any daycare in the first year of life.
Smoke exposure[8]	Generated, binary	Positive if mother smoked in pregnancy or if there was ever a smoker living in the house on any questionnaire. Negative, if there is no evidence of smoking at any time point (no or missing)
Sex[9]	Raw, binary	Assigned male or female at birth

Breastfeeding duration[8]	Generated, categorical	No breastfeeding, <1 week, 1 wk – 3 months, 3-6 months, 6-12 months, >12 months
Family history of asthma[8]	Generated, binary	Positive if mother, father or sibling has asthma
Pet ownership at birth[10]	Generated, binary	Positive, if the family had a pet with hair at randomisation (e.g., dog, cat, rabbit, cow)
Delivery mode[8]	Raw, binary	Vaginal or caesarean birth
Season of birth[11]	Generated, categorical	Categories of birth based on season (Spring (Sept – Oct), Summer (Nov - Jan), Autumn (Feb – May), Winter (Jun – Aug)
Maternal age at birth of participant[12]	Raw, continuous	Age in years
Paternal age at birth of participant[13]	Raw, continuous	Age in years
Low birthweight[14]	Generated, binary	Low birthweight is <2500g
Timing of recruitment	Generated, categorical	Categories determined based on date of recruitment.
Evidence of asthma[15]	Generated, binary	Positive if parent reported asthma or wheeze disturbing sleep or exercise at 36 or 48 months or said yes on the follow-up questionnaire. Negative if completed the 36 and 48 month questionnaires and did not report those symptoms or said no on the follow-up questionnaire.
Hospitalisation for bronchiolitis in the first year of life[16]	Generated, binary	Positive if parent reports hospitalisation for bronchiolitis in the first year of life. Negative if all 3 month, 6 month, 9 month and 12 month questionnaires were complete and no hospitalisation for bronchiolitis was reported.
Atopy (eczema[15,17,18] or atopic sensitization[15,19]) at one year of age	Generated, binary	Positive if positive for either eczema or atopic sensitisation at one year of age. Negative if negative for both eczema and atopic sensitisation at one year of age.

5.2.4. SUBGROUP ANALYSIS

Additional adjusted models will be estimated to explore the potential heterogeneity of the effect of the intervention. Each model will include as covariates the mode of birth stratification factor, and an interaction term estimating the interaction between the intervention and the sub-group variable (listed below). Where the models provide evidence that the intervention varies between sub-groups, the intervention effect in each sub-group and their 95% confidence intervals will be

presented, together with the p-value for the intervention-by-subgroup interaction, as a guide to the strength of the evidence for an interaction. All sub-group analysis will be conducted on the imputed data.

Sub-groups are variables that are known or suspected risk factors for asthma in childhood that are also known or suspected to influence the immune response to BCG vaccination. Sub-group analysis will include:

1. Either parent has a history of asthma[8]
2. Sex[9]
3. Maternal history of BCG vaccination

5.3. PRIMARY DATA ANALYSES

The percentage of 'asthma ever' reported at five years will be presented separately for children in the treatment and non-treatment groups. Comparison between the treatment arms will be estimated using binary regression adjusted for the randomisation stratification factor of birth mode. Results will be presented as adjusted risk difference and its 95% confidence interval (CI). The analysis will be presented as ITT with multiple imputation as described above.

The two other stratification factors used for randomisation (site and plurality) will not be included in adjustments because it is already known from Part 1 that 98.3% of infants were singleton and 95.2% were from the same site [5,6].

As a sensitivity analysis, the same binary regression model will be re-run excluding the 7-10 year follow-up questionnaire data.

5.4. SECONDARY DATA ANALYSES

For each secondary outcome, the percentage of study participants with the outcome will be presented separately for children in the treatment and non-treatment groups. Comparison between the treatment arms will be estimated using binary regression adjusted for the randomisation stratification factor of birth mode. Results will be presented as adjusted risk difference and its 95% confidence interval (CI).

Intention to treat analysis with multiple imputation will be used for all the secondary outcomes.

5.5. ANALYSIS OF OTHER OUTCOMES

For other outcome variables, the percentage of study participants with the outcome will be presented separately for children in the treatment and non-treatment groups. No formal statistical comparisons will be made.

6. REFERENCES

1. Messina NL, Gardiner K, Donath S, et al. Study protocol for the Melbourne Infant Study: BCG for Allergy and Infection Reduction (MIS BAIR), a randomised controlled trial to determine the non-specific effects of neonatal BCG vaccination in a low-mortality setting. *BMJ Open*. Dec 15 2019;9(12):e032844. doi:10.1136/bmjopen-2019-032844
2. Australian Institute of Health and Welfare. *Asthma in Australia 2011: with a focus chapter on chronic obstructive pulmonary disease*. 2011. https://www.aihw.gov.au/reports/chronic-respiratory-conditions/asthma-in-australia-2011-with-chapter-on-copd
3. Asher MI, Keil U, Anderson HR, et al. International Study of Asthma and Allergies in Childhood (ISAAC): rationale and methods. *Eur Respir J*. Mar 1995;8(3):483-91. doi:10.1183/09031936.95.08030483
4. Haktanir Abul M, Phipatanakul W. Severe asthma in children: Evaluation and management. *Allergol Int*. Apr 2019;68(2):150-157. doi:10.1016/j.alit.2018.11.007
5. Messina NL, Pittet LF, Gardiner K, et al. Neonatal Bacille Calmette-Guérin Vaccination and Infections in the First Year of Life: The MIS BAIR Randomized Controlled Trial. *J Infect Dis*. Oct 13 2021;224(7):1115-1127. doi:10.1093/infdis/jiab306
6. Pittet LF, Messina NL, Gardiner K, et al. Prevention of infant eczema by neonatal Bacillus Calmette-Guérin vaccination: The MIS BAIR randomized controlled trial. *Allergy*. Mar 2022;77(3):956-965. doi:10.1111/all.15022
7. Ochoa Sangrador C, Vázquez Blanco A. Day-care center attendance and risk of Asthma-A systematic review. *Allergol Immunopathol (Madr)*. Nov-Dec 2018;46(6):578-584. doi:10.1016/j.aller.2018.03.006
8. Castro-Rodriguez JA, Forno E, Rodriguez-Martinez CE, Celedón JC. Risk and Protective Factors for Childhood Asthma: What Is the Evidence? *J Allergy Clin Immunol Pract*. Nov-Dec 2016;4(6):1111-1122. doi:10.1016/j.jaip.2016.05.003
9. Naeem A, Silveyra P. Sex Differences in Paediatric and Adult Asthma. *Eur Med J (Chelmsf)*. Jun 2019;4(2):27-35.
10. Gergen PJ, Mitchell HE, Calatroni A, et al. Sensitization and Exposure to Pets: The Effect on Asthma Morbidity in the US Population. *J Allergy Clin Immunol Pract*. Jan-Feb 2018;6(1):101-107.e2. doi:10.1016/j.jaip.2017.05.019
11. Almqvist C, Ekberg S, Rhedin S, Fang F, Fall T, Lundholm C. Season of birth, childhood asthma and allergy in a nationwide cohort-Mediation through lower respiratory infections. *Clin Exp Allergy*. Feb 2020;50(2):222-230. doi:10.1111/cea.13542
12. Laerum BN, Svanes C, Wentzel-Larsen T, et al. Young maternal age at delivery is associated with asthma in adult offspring. *Respir Med*. Jul 2007;101(7):1431-8. doi:10.1016/j.rmed.2007.01.020
13. Thomsen AML, Ehrenstein V, Riis AH, Toft G, Mikkelsen EM, Olsen J. The potential impact of paternal age on risk of asthma in childhood: a study within the Danish National Birth Cohort. *Respir Med*. Apr 2018;137:30-34. doi:10.1016/j.rmed.2018.01.016
14. Xu XF, Li YJ, Sheng YJ, Liu JL, Tang LF, Chen ZM. Effect of low birth weight on childhood asthma: a meta-analysis. *BMC Pediatr*. Oct 23 2014;14:275. doi:10.1186/1471-2431-14-275
15. Biagini Myers JM, Schauberger E, He H, et al. A Pediatric Asthma Risk Score to better predict asthma development in young children. *J Allergy Clin Immunol*. May 2019;143(5):1803-1810.e2. doi:10.1016/j.jaci.2018.09.037
16. Beigelman A, Bacharier LB. The role of early life viral bronchiolitis in the inception of asthma. *Curr Opin Allergy Clin Immunol*. Apr 2013;13(2):211-6. doi:10.1097/ACI.0b013e32835eb6ef
17. Amat F, Soria A, Tallon P, et al. New insights into the phenotypes of atopic dermatitis linked with allergies and asthma in children: An overview. *Clin Exp Allergy*. Aug 2018;48(8):919-934. doi:10.1111/cea.13156

18. Yaneva M, Darlenski R. The link between atopic dermatitis and asthma- immunological imbalance and beyond. *Asthma Res Pract*. Dec 15 2021;7(1):16. doi:10.1186/s40733-021-00082-0
19. Vermeulen EM, Koplin JJ, Dharmage SC, et al. Food Allergy Is an Important Risk Factor for Childhood Asthma, Irrespective of Whether It Resolves. *J Allergy Clin Immunol Pract*. Jul-Aug 2018;6(4):1336-1341.e3. doi:10.1016/j.jaip.2017.10.019

7. CHIEF INVESTIGATOR AGREEMENT

Name	Signature	Date
Prof Nigel Curtis	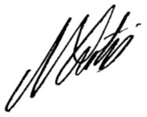	16 Jan 2024
A/Prof Susan Donath	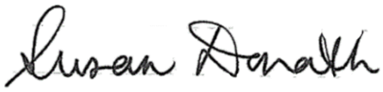	16th January 2024
